# Outdoor Physical Activity in an Air Polluted Environment and Its Effect on the Cardiovascular System—A Systematic Review

**DOI:** 10.3390/ijerph191710547

**Published:** 2022-08-24

**Authors:** Taruna Juneja Gandhi, Priyanka Rani Garg, Kauma Kurian, Jonas Bjurgert, Sirazul Ameen Sahariah, Sunil Mehra, Gayatri Vishwakarma

**Affiliations:** 1 MAMTA Health Institute for Mother and Child, B-5, Greater Kailash Enclave II, New Delhi 110048, India; 2 Lund University, Box 117, SE-221 00 Lund, Sweden; 3 Indian Spinal Injuries Centre Sector-C, Vasant Kunj, New Delhi 110070, India

**Keywords:** air pollutants, physical activity, cardiovascular disease, outdoor physical activity, environment and public health, exercise

## Abstract

Air pollution is a global public health threat. Evidence suggests that increased air pollution leads to increased cardiovascular morbidity and mortality. The aim of this review was to systematically review and synthesize scientific evidence to understand the effect of performing outdoor physical activity (PA) in a polluted environment on cardiovascular outcomes. This review was developed and reported in accordance with the PRISMA guidelines. Electronic searches in Embase, Web of Science, and PubMed were undertaken through March 2021 initially, and later updated through to 31st January 2022, for observational studies published in peer-reviewed journals that report cardiovascular mortality or morbidity due to outdoor PA in air polluted environment. These searches yielded 10,840 citations. Two reviewers independently reviewed each citation for its eligibility. Seven studies were found to be eligible. Of these, five were cohort studies and two were cross-sectional studies. Pollutants measured in the selected studies were Particulate Matter (PM)—PM10, PM2.5, nitrogen oxides (NO_x_), and ozone (O_3_). The most common study outcome was myocardial infarction, followed by cardiovascular mortality, hypertension and heart rate variability. Six studies emphasized that the PA has beneficial effects on cardiovascular outcomes, though air pollutants attenuate this effect to an extent. Two studies showed that walking, even in the polluted environment, significantly reduced the heart rate and heart rate variability indices. The beneficial effects of outdoor PA outweigh the harmful effects of air pollution on cardiovascular health, though the benefits reduce to an extent when PA is carried out in a polluted environment. Because a limited number of studies (*n* = 7) were eligible for inclusion, the review further emphasizes the critical need for more primary studies that differentiate between outdoor and indoor PA and its effect on cardiovascular health.

## 1. Introduction

Air pollution is a major global public health issue affecting the daily lives of people and predisposing them to a number of health issues. Evidence suggests that exposure to air pollution affects multiple organ systems and functions, including cardiovascular, respiratory, cognitive, and sleeping patterns; increasing the incidence of all-causes mortality. According to the World Health Organization (WHO), ambient air pollution was responsible for around 4.2 million premature deaths worldwide per year in 2016 in both urban and rural areas, mainly due to the exposure to particulate matter 2.5 (PM2.5). Around 91% of these premature deaths were reported in low and middle-income countries (LMICs), with the greatest burden in the WHO South-East Asian and Western Pacific regions [1]. It is known that ambient air pollution, frequently found in urban areas, is a dynamic and a complex mixture of both man-made pollutants and natural sources [2]. The six common ambient air pollutants are particulate matter (PM2.5, PM10), ozone (O_3_), sulphur dioxide, initrogen oxides (NO_x_), carbon monoxide, and lead [2]. Exposure to outdoor air pollution is the ninth leading risk factor for mortality; outdoor air pollution is responsible for 3.2 million deaths every year [3].

Various research studies have demonstrated that increased air pollution leads to increased cardiovascular mortality, including a higher risk of arteriosclerosis, ischemic heart disease (IHD), heart failure, and ischemic/thrombotic stroke [4,5,6]. Short-term exposure to air pollution has been associated with an increased risk of myocardial infarction (MI), stroke and acute heart failure. Recent research has found evidence that air pollutants, such as PM10 or PM2.5, are linked to increased incidence and mortality of cardiovascular diseases (CVDs) [7,8,9]. CVDs continue to be the main cause of death around the world. According to the Global Burden of Disease (GBD) study, an estimated 17.6 million global deaths were attributable to CVDs between 2006 and 2016, which contributed to more deaths than any other disease. Ample evidence is available related to protective and risk factors of CVDs [10].

Physical activity (PA) has long been recognized for its health advantages, which include lower all-cause mortality and preventive effects pertaining to CVDs [11,12]. However, in a polluted environment, outdoor PA may be associated with poor health outcomes or increased risk of CVDs. Despite the health benefits of PA, doing it in a polluted environment may result in adverse health impacts due to the increased intake of air pollutants [13]. The trade-off between the health advantages related to greater PA and the potential adverse consequences due to increased exposure to air pollution while undertaking outdoor PA is still debated [7].

Evidence suggests that PA increases the exposure to air pollutants by increasing the inhaled dose leading to higher deposition of the inhaled particles in lungs and higher minute ventilation (exercise-induced) [14]. In vulnerable individuals, PM2.5 has been linked to an increased risk of MI, stroke, arrhythmia, and exacerbation of heart failure within hours to days of exposure. Several recent studies have found that living in areas with higher long-term average PM levels increases the risk of cardiovascular morbidity and mortality [15]. The concentration of fine PM and the rate of CVDs were studied among 50 million people residing in the 20 largest cities and metropolitan areas in the United States (The National Morbidity, Mortality and Air Pollution Study, NMMAPS). According to this research, a 10 μg/m^3^ rise in PM10 on the day preceding death was found to be related to a 0.68% increase in cardiopulmonary mortality [16,17,18]. PM10 research has also revealed a link between air pollution and CVDs such as heart disease and stroke. According to a study, every 10 ug/m^3^ rise in PM10 levels results in an increase in hospitalizations caused due to Ischemic Heart Disease (IHD) (0.7%) and Congestive Heart Failure (CHF) (0.8%) [19].

The combined effects of PA and air pollution on CVDs have piqued the interest of researchers [20]. To date, as per our knowledge, no systematic review has examined the effects of PA in an outdoor polluted environment on incident and recurrent CVDs. With the ever-increasing pollution levels and an impending threat of climate change, there is an urgent need to understand this missing link between outdoor PA, air pollution and CVDs. The purpose of this study was to systematically review and synthesize the available scientific evidence to understand the effect of engaging in outdoor PA in a polluted environment on cardiovascular outcomes.

## 2. Materials and Methods

This systematic review was developed and reported in accordance with the Preferred Reporting Items for Systematic Review and Meta-analysis (PRISMA) guidelines which are evidence-based and consist of a minimum set of items focusing on the reporting of reviews evaluating various types of research [21]. Before the formal screening of search results, the protocol for this study was registered in PROSPERO (http://www.crd.york.ac.uk/PROSPERO/ (accessed on 10 May 2022)) under the registration number CRD42021256354.

### 2.1. Review Characteristics

Types of studies: The studies were selected based on the following inclusion and exclusion criteria. Observational studies published in a peer-reviewed journal till 31 January 2022 that reported cardiovascular mortality or morbidity as a result of outdoor PA in a polluted environment were considered for inclusion. Studies with human subjects and published in the English language were considered in this systematic review. This review excluded all reviews, meta-analyses, letters, editorials, commentaries, conference papers, and reports that considered PAs such as farming, commuting, and occupational-related activities, as well as indoor exercises.

Type of participants (Population): People over the age of 18 years who participated in outdoor PA such as exercise, running, walking, jogging, cycling, and other similar activities.

Type of exposure (Exposure): Pollutants, including black carbon, carbon monoxide, lead, nitrogen dioxide, nitrogen oxide, ozone, PM, sulphur dioxide, and sulphur oxide that contribute to outdoor air pollution.

Type of controls (Comparison): Population/people who engaged in exercise/s in clean air or filtered air, or less air polluted environments.

Type of outcomes (Outcomes): Any cardiovascular-related morbidity and mortality.

### 2.2. Literature Search

Information sources: Electronic databases such as Embase, Web of Science, and PubMed were searched to identify the relevant articles.

Search strategy: A search strategy was developed using the indexed and free-text terms of population, exposure, comparison, and outcome (PECO) statement, for each of the databases searched for the systematic review. The following PECO terms were used in conjunction with Boolean connectors: exercises, games, recreational, jogging, leisure activities, running, sports, and walking (for population); air pollution, air pollutants, carbon black, carbon monoxide, lead, nitrogen dioxide, nitrogen oxides, ozone, particulate matter, sulfur dioxide, sulfur oxides, ambient air pollution*, outdoor air pollut*, PM10, PM2.5, Cycl*, outdoor physical activi*, and physical activit* (for exposure); and, cardiovascular diseases, cardiovascular system, heart diseases, morbidity, mortality, vascular diseases, adverse effect*, cardiac event*, cardiac outcome*, cardiovascular disorder*, cardiovascular event*, cardiovascular outcome*, circulatory system, CVD*, vascular event*, and vascular outcome* (for outcome). The final search string used for PubMed can be found in the Supplementary File (Table S1).

### 2.3. Article Selection

All the records were screened separately by two independent reviewers for its eligibility. The titles were screened first, followed by the abstracts, and finally the full text of each article was reviewed thoroughly. A study moved on to the next stage of screening only if both the reviewers agreed that it must be included. Only studies that were approved by at least two authors were included in the full-text screening. To reach a consensus, a third reviewer arbitrated if there were any disagreements between the two reviewers. T.J.G., P.R.G., K.K. and J.B. screened the studies; S.A.S., S.M. and G.V. arbitrated the disagreements. All the excluded studies were given a definite reason for their exclusion. The result from all the databases (10,840 citations) were directly imported into the EndNote software. There were 622 duplicates and 41 citations with no content, which were excluded from the review. Following the removal of duplicates and empty citations, a total of 10,117 studies remained for title and abstract screening.

### 2.4. Data Extraction

Following an extensive literature review and discussion with subject and methodological experts, a standardized, pre-tested data extraction form was developed. The draft data extraction form was then piloted and tested independently by two experts. Changes were made in response to their comments and suggestions on the form. Data were extracted using Microsoft Excel 2016 by one reviewer and verified by two experts. Categories under which data were extracted included (a) study characteristics (b) methodological characteristics (c) details of PA (d) exposure characteristics (e) outcome/s of the study.

### 2.5. Quality Assessment

After completing the full-text screening, all the selected research papers were appraised for quality using the Joanna Briggs Institute Quality Appraisal Tool (available at: https://jbi.global/critical-appraisal-tools). The JBI Critical Appraisal Tool has a separate checklist for each of the study designs, which were used for various types of studies included in the current systematic review [22]. The JBI critically views what counts as evidence and the methodology used to synthesize different varieties of evidence. The purpose of the JBI appraisal tool is to assess the quality of the methodology used in a study. The tool determines the extent to which a study has addressed the possibility of bias in its design, conduct and analysis. Two reviewers (out of K.K., P.R. G or T.J.G) independently assessed the quality of evidence of each article and arbitrated the disagreements.

### 2.6. Data Synthesis

A narrative synthesis was undertaken for the research studies included in the review. The synthesis summarized various cardiovascular outcomes associated with outdoor exercise in a polluted environment. After consultation with subject and methodological experts, the details regarding the pollutants, PA, and cardiovascular outcomes were carefully extracted. Then, the outcomes were grouped according to the levels of pollution and pollutants, and according to levels of outdoor PA followed by a narrative synthesis of each reported outcome. The characteristics of the included studies are presented in the form of a table along with a narrative summary (Table 1). Additionally, the impact of study quality, strengths and limitations of the included studies have also been discussed in the narrative summary.

## 3. Results

### 3.1. Search Result

The database search yielded a total of 10,840 citations, out of which, 1986 citations were found in Embase; 2303 in PubMed; and 6551 in Web of Science. After removing 663 duplicates and empty citations, 10,177 records were left for the title and abstract screening. In total, 10,076 records were excluded during the title and abstract screening stage, and 101 articles remained for full-text screening. In the full-text screening stage, 91 articles were excluded. 

**Figure 1:**
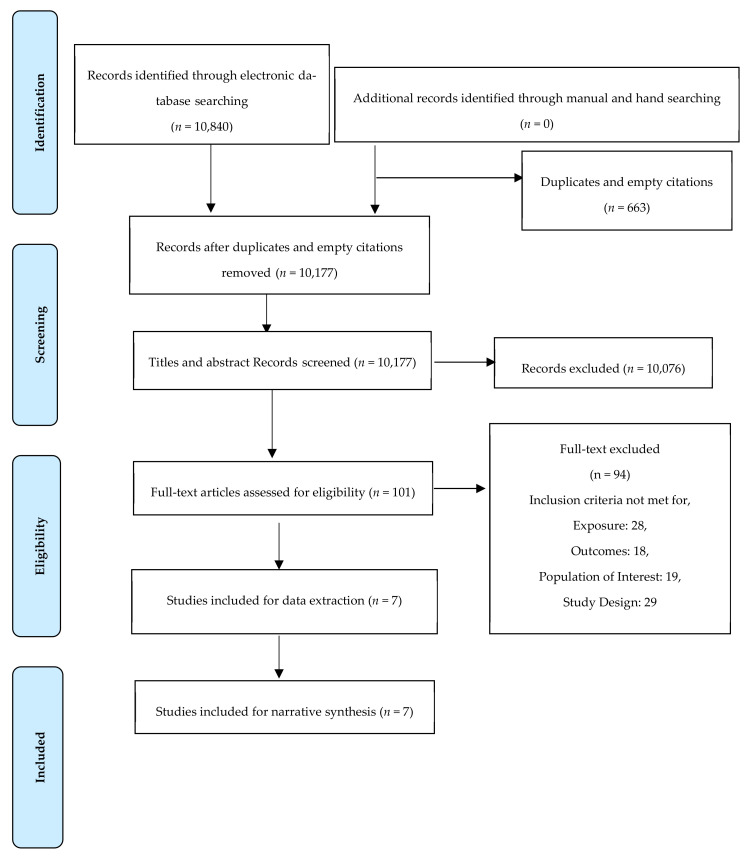
The PRISMA flow chart illustrating search and screening results.

Out of the remaining 9 studies, two studies [30,31] did not make it clear whether the PA considered in the studies was performed outdoor or indoor or both. To clarify this issue, email enquiries were sent to the respective authors of the studies. However, no response was received from the authors and these studies were finally eliminated from the current systematic review. Finally, 7 studies were included for data extraction and quality appraisal. Further, only a narrative synthesis could be carried out due to the heterogeneity of the data in the included studies. Figure 1 depicts the PRISMA flow diagram for the article selection process and the reasons for article exclusion at various levels.

### 3.2. Quality Appraisal

The quality appraisal was done for all the studies included after the full-text screening. The quality of evidence of each article included in the review was assessed independently by two reviewers using the Joanna Briggs Institute (JBI) Quality Appraisal Tool [22]. The overall quality of most of the articles was good as the criteria of the respective checklists used for appraising the quality of the research studies was fulfilled (Supplementary File: Table S2). There was no disagreement between the two reviewers regarding the inclusion of papers based on the quality checks, and a total of 7 papers were eventually included in the current systematic review.

### 3.3. Characteristics of Included Articles

Seven studies met the inclusion criteria of the systematic review and the study characteristics of all the 7 papers are given in Table 1. Five studies were cohort studies [23,24,26,27,29] and 2 were cross-sectional studies [25,28]. Four studies were conducted in the developed countries (2 each in the United States [24,25] and Denmark [23,26]), and three were done in developing countries (2 in China [27,29] and one in Brazil [28]). Four studies [23,24,26,27] utilized the data from the already existing cohorts i.e., Danish Diet, Cancer, and Health cohort [23,26]; Nurses’ Health Study [24]; and Prediction for Atherosclerotic Cardiovascular Disease Risk in China project (China-PAR) [27]; one study utilized the data from the Regional Travel Survey [25].

The sample size ranged from 120 [28,29] to 104,990 [24], and the total sample size of this systematic review is 315,342. The age distribution of the participants ranged from 18 to 45 [28]; 19 to 65 [23]; 50–65 years [23,26]; 30–55 years [24]; 21–54 years [25]; 19–24 years [29] in studies. It should be noted that three studies [25,28,29] considered the young population. Commonly measured pollutants in the studies were PM10 [29], PM2.5 [24,25,27,29], Nitrogen Oxides (NO_x_) [23,25,26,28] and Ozone (O_3_) [25,28].

### 3.4. Summary of Exposures

The most commonly measured pollutants were PM2.5 [24,25,27,29] and NO_2_ [23,25,26,28], both measured in four out of seven studies. None of the seven studies included all the above-mentioned pollutants together. The most common study outcome was MI, considered in three studies [24,26,27], followed by cardiovascular mortality in two studies [23,27], while hypertension [26], heart rate variability (HRV) [29], and IHD [25] were assessed in one study each. One study considered total mortality [24], while another study considered total mortality as well as diabetes-related mortality [23]. Table S2 (in Supplementary File) provides an overview of the summary of findings for each article included in the narrative synthesis.

### 3.5. Description of Physical Activity and Its Measurements

The main outdoor PA mentioned in the studies were walking [23,24,25,26,27,29]; cycling [23,24,25,26,27]; sports [23,25,26]; gymnastics [26]; badminton [26]; gardening [23,26]; jogging [24]; running [24,26]; swimming [24,26]; tennis [24]; aerobic physical activities [24,28]; lawn mowing [24]. The measurement of PA varied from study to study. Studies used Metabolic Equivalent of Task (MET) score [24,28]; self-administered interviewer-checked questionnaire [26,27,28]; objective monitoring; and post-Census Regional Travel Survey data [25]. 

A study by Anderson, et al. [23] assessed the PA by a self-administered, interviewer-checked questionnaire in which the leisure time PA was reported as hours per week spent on sports, cycling, gardening, and walking. Data were collected separately for winters and summers, and the two values were averaged; being active implied at least half an hour spent on a specific activity per week.

Elliott, et al. 2020 [24] used an ‘MET score’ to assess PA and calculated the overall PA in MET-hours per week by summing the MET-hours per week across all activities. Participants reported spending time on each of the PAs, which were divided into several categories, ranging from 0 min to ≥11 h per week. Time spent on each PA per week was multiplied by the MET score for each activity to calculate MET-hours per week, which accounted for frequency, duration, and intensity of PA.

The study by Kubesch, et al. 2018 [26] collected the data on PA by a self-administered, interviewer-checked questionnaire and it was reported as hours per week spent on various outdoor PAs. The data were collected in the winter and summer, and the two values were averaged. The majority (99.2%) of the study participants reported being involved in one of the four PAs included in the study. Of all the participants, 54% participated in sports, 68% in cycling, 74% in gardening, and 93% in walking. Cohort members who participated in PAs spent on an average 2.4 h/week participating in sports, 3.2 h/week cycling, 3.0 h/week gardening, and 4.3 h/week walking. Liu 2015 [29] conducted a study among students at Taipei University, Taiwan. In total, 120 young, healthy students were enrolled in the study and used different commuting modes. Each student engaged in 1 h morning commuting-walking along with three other commuting methods—electrically powered subway, gas-powered bus, and gasoline-powered car.

Another study by Hankey, et al. [25] used the Regional Travel Survey from 2001 to estimate physical inactivity. The survey also included a geocoded time-activity diary, which recorded self-reported activities and travel. Each participant’s one-day activity record was multiplied by 7 to get an estimate of weekly minutes spent on PA. This method assumed that PA was consistent throughout the week. An additional weekend survey supplement was carried out for a small number of respondents (13%, *n* = 5104) and population-average levels of PA were calculated. These levels were found to be similar (<15% difference) between weekdays and weekends (11 vs. 12 min/day, respectively).

Elliott, et al. [24] considered MET-hours per week from: walking alone; vigorous-intensity activities (jogging, running, biking, swimming, and playing tennis); low- or moderate-intensity activities; and PAs likely to be performed outside (walking, running, lawn mowing). In the study by Hankey, et al. [25] the total PA was broken down into active transportation, such as walking, bicycling, and recreational activities, such as sports and working out in the gym. The average amount of self-reported PA was 77 min per week. According to the survey, 83.5% of participants were found to be inactive (0 min per week), 5.6% were insufficiently active (1–150 min per week), and 10.9% were active (>150 min per week).

For the study by Lin, et al., the information on commuting mode was collected by asking “what kind of commuting mode do you take to and from work?”, and the options given were “A. Walking; B. Cycling; C. Public transportation; D. Driving (Car or motorcycle); E. No need to commute (i.e., Working at home)”. Commuting modes were categorized into three types: non-active (including public transportation, driving, and no need to commute), walking and cycling. Non-active commuting mode served as a reference in this study [27].

Another study by Marmett, et al. [28], considered the ideal time for exercise to be >150 min/week, based on the WHO recommendation of 150–300 min of moderate-intensity aerobic PA for adults. The data were collected using the International Physical Activity Questionnaire (IPAQ). The PA level was derived from the IPAQ for population use, which included the frequency and duration of activities as walking, moderate and vigorous intensities. The product of PA intensity (MET—Metabolic Equivalent of Task) and duration (h) was calculated as the volume of the activity (MET-hours) [28].

### 3.6. Pollution Exposure Assessment

The most commonly examined pollutant was PM2.5 (4 studies) [24,25,27,29] and NO_2_ (4 studies) [23,25,26,28]. Other pollutants reviewed in the included studies were PM10 [29], Ozone [25,28], and NO_3_ [25]. One of the studies reviewed the effects of PM2.5, NO_x_, and O_3_ [25]; another assessed the impact of both PM2.5 and PM10 [29]; while another three studies reported the individual effects of NO_2_ [23,26] and PM2.5 [24] each. Most of the studies (5 out of 7) used the nationally available data for estimating the concentration of pollutants at the residential addresses [23,24,25,26,27]. 

The selected studies adopted different approaches to assess high resolution (up to the residential address level) exposure estimates for the respective populations. The methodologies of measuring pollutant levels varied from a personal dust monitor [29] to using the country’s air surveillance systems [23,24,25,26], as mentioned in Table 2. The studies utilized different countries’ monitoring data [23,24,25,26], land use regression approaches [25], satellite-based spatiotemporal models [27], and spatiotemporal prediction models [24] to improve predictions of air pollutants.

Kubesch, et al. [26] estimated the concentration of NO_2_ using the Danish AirGIS Dispersion Modelling System. This system is based on geographical information system (GIS) and provides estimates of Traffic-Related Air Pollution (TRAP) with temporal (1-year averages) and spatial (address-level) resolution. The average exposure to TRAP in general, and during exercise were estimated through a proxy indicator ‘the annual mean concentrations of NO_2_′ at residential addresses of each cohort participant. The mean NO_2_ concentration was categorized as low (25th percentile of exposure range: <14.3 lg/m^3^), medium (≥14.3–21.0 lg/m^3^), and high (upper 25th percentile of exposure range: ≥21.0 lg/m^3^) exposure to NO_2_, which corresponded to each participant’s recruitment year and therewith to the same year for which participants reported PAs. In Andersen, et al. [23], outdoor concentration of nitrogen dioxide (NO_2_) was calculated at the residential addresses of each cohort member with the Danish AirGIS dispersion modelling system. The mean annual concentrations of NO2 at residential addresses of each cohort participant since 1971 until the end of follow-up was used as a proxy of average exposure to TRAP during exercise. The indicator was defined as high versus moderate/low NO_2_ exposure separated by the 75th percentile of exposure ranges in the cohort (≥ vs. <19.0 μg/m^3^).

Elliott, et al. [24] calculated the exposure of PM2.5 at residential addresses (updated every two years) using spatiotemporal prediction models available in United States between January 1988 and December 2007. The study used the monthly average PM2.5 and PM10 monitoring data from the U.S. Environmental Protection Agency’s (USEPA) Air Quality System. The generalized additive mixed models incorporated geospatial predictors (road network data, residential and urban land use, density of PM2.5 and PM10 point-sources, and elevation data) and monthly average meteorological data (wind speed, temperature, precipitation). The 24-month moving averages were calculated for each questionnaire cycle as a measure of long-term exposures. The participants were excluded for the corresponding questionnaire cycle in the analyses if the 24-month average PM2.5 were missing.

Hankey, et al. [25] investigated the built environment’s association with air pollution, physical inactivity, and estimated attributable health risks. The study modelled and measured the estimates of outdoor air pollution and their variability in space and time based on the monitoring data from the U.S. Environmental Protection Agency (USEPA) 2010 for PM2.5, NO_x_, and O_3_ in 2001 (primary estimate for air pollution exposure). The study included concentrations of pollutants (inverse-distance weighted average of the nearest three monitors) to the home location of each respondent. As each pollutant had several monitoring stations, the study estimated the annual average of daily one-hour concentrations for O_3_ and the annual-average concentrations for PM2.5 and NO_x_ at each survey participant’s residence. The study calculated three built environment variables to represent neighbourhood type, including population density, intersection density and land use mix [25]. In Liu, et al. [29] panel study and one-hour continuous air pollution monitoring were performed during measurement of air pollutants for each participant. One-minute mass concentrations of PM10 and PM2.5, temperature, and relative humidity were measured and recorded continuously using a personal dust monitor. Total volatile organic compounds (TVOCs) were measured continuously using a TVOCs monitor with the detection limit for TVOCs concentration of 1ppb.

In Lin, et al., satellite-based spatiotemporal models were applied to estimate environmental fine particulate matter (PM2.5) exposure levels from 2000 to 2015. An ensemble machine learning model was developed to estimate ground PM2.5 concentration of China based on the Aerosol Optical Depth (AOD) retrieved from the National Aeronautics and Space Administration (NASA) Aqua and Terra satellites, meteorology, land use information and population density data. The model was verified using the ground PM2.5 measurement results of the China Environmental Monitoring Center. With the satellite-based spatiotemporal model, the monthly PM2.5 concentrations in China was obtained from the year 2000 to 2015. The residential address of each participant was geocoded as grid cells according to latitude and longitude data. The average monthly PM2.5 concentrations from 2007 to 2008 survey to 2015 were calculated for each participant [27].

Marmett, et al., used personal exposure samplers of O_3_ and NO_2_ to measure the concentration of pollutants through passive monitoring. On the data collection day, all subjects received two diffusion tubes, one to monitor O_3_ concentration and other for measuring NO_2_ concentration, together with the instructions for correct management [28].

### 3.7. Description of Outcomes and Measurements (Including Findings of the Studies)

Most common outcomes in the studies included in the systematic review were hypertension [23,26], MI [23,26,27], and stroke [23,26,27]. CVD-related mortality was assessed by two studies [23,27] while fatal or non-fatal CHD events and CHD-related mortality were assessed in one study [27]. Heart rate variability (HRV) indices were measured in one study [29] while another study used lipid accumulation product (LAP) index that considered waist circumference and blood triglyceride levels (important CVD markers) [28].

Study by Kubesch, et al. [26] reported the long-term benefits of PA. They found that engaging in PA prevented the development of MI and outweighed the risks associated with exposure to air pollution. The study concluded that the benefits of PA in reducing the risk of MI are not reduced by exposure to air pollution in metropolitan cities with high air pollution levels. Intermittent PA attenuates the TRAP-related increases in systolic blood pressure (SBP), however, this is not true for PM10 and PM coarse which potentiate these increases. In low-TRAP environments, intermittent PA has stronger beneficial effects on SBP than in the high-TRAP environments. The panel study by Liu et al. [29] compared the TRAP levels with HRV indices between four different commuting modes—an electrically powered subway, a gas-powered bus, a gasoline-powered car, and walking. It was found that walking was significantly linked to lower HRV indices due to the positive correlation between the walking mode and exposure to air pollution (PM10, PM2.5 and TVOCs) as compared with other commuting modes. The subjects in the walking mode had significantly reduced SDNN and r-MSSD values (23 and 37% lower than the subway mode, 17 and 19% lower than the car mode and 12 and 21% lower than in the bus mode, respectively) due to PM2.5. They found that it was only PM2.5 that was significantly associated with decreased SDNN and r-MSSD, for PM2.5 and PM10; PM2.5 and TVOCs mixed-models. It was concluded that traffic-related PM2.5 was associated with autonomic alteration and commuting modes can modify the effects of PM2.5 on HRV indices among young healthy subjects.

Andersen, et al., 2015 iterated that an inverse association exists between engaging in PA and cardiovascular mortality which was not modified by pollutants like NO_2_. This inverse association was stronger between cycling and gardening and cardiovascular mortality. Exposure to high levels of TRAP did not modify this association, indicating beneficial effects of PA on cardiovascular mortality [23]. Hankey, et al., 2012 investigated the relationship between built environment (in terms of high vs. low walkability) with air pollution, physical inactivity and estimated IHD mortality. They found differences between neighbourhoods for the estimated IHD mortality caused due to air pollution—9 more IHD deaths/100,000/year for PM2.5 and 3 fewer IHD deaths100,000/year for O_3_ in high vs. low-walkability neighbourhoods. This suggests the offsetting of the positive effects of PA on cardiovascular health due to adverse effects of air pollution exposure [25].

In a study by Lin et al. 2021 on three cohorts of Chinese adults, the authors investigated the association between active commuting (cycling or walking) and cardiovascular diseases (CVDs), mortality and life expectancy. The study evaluated the modification effect of fine particulate matter (PM2.5) exposure on these associations. It was found that active commuting had a protective effect on CV health and resulted in lower risk of CVDs, all-cause mortality, and longer life expectancy but under ambient settings of lower PM2.5 level. A study by Marmett, et al., 2022 evaluated the effects of O_3_ and NO_2_ exposure on cardiorespiratory fitness, lipid accumulation product (LAP), and environmental health risk during the daily routine of physically active adults who engage in PA in outdoor and indoor environments. The study found that physically active individuals exhibited a lower risk of developing cardiovascular and metabolic diseases irrespective of the higher O_3_ concentration exposure during exercise.

## 4. Discussion

There is ample evidence that regular PA has a beneficial effect on cardiovascular health. We also know that an outdoor polluted environment is harmful to the cardiovascular health of individuals and populations at large. However, less information is available regarding the effects of engaging in outdoor exercise/s in a polluted environment on cardiovascular health. This systematic review was designed to assess the joint effects of outdoor PA and air pollution on cardiovascular health. In total, 101 studies were included for full article screening, but seven studies met the inclusion criteria during the data extraction stage. Further, due to the difference in the types of air pollution exposure, types of exercise performed, and cardiovascular health indicators measured in different studies, the effect of combined outdoor PA and air pollution exposure on cardiovascular health could not be quantified.

Most of the studies (6/7) included in this systematic review emphasized the favourable effect of PA on the cardiovascular health of the population [23,24,25,26]. The beneficial effects of PA outweighed the harmful effects of outdoor air pollutants although the presence of air pollutants attenuate the beneficial effect of PA to an extent. Most of the studies included in the systematic review highlighted how the positive effects of PA performed by individuals are reduced to an extent when they are exposed to ambient air pollution. We critically reviewed the strengths and weaknesses of each included study to gain an analytical understanding of their outcomes in light of the pollutant/s considered in the studies.

A longitudinal study by Elliot, et al. [26] followed up participants for 20 years; 6074 incident CVD cases and 9827 deaths were documented. The study observed no multiplicative interaction between long-term PM2.5 exposure and physical activity; higher physical activity (overall, walking, vigorous activity) was strongly associated with lower CVD risk and overall mortality at all levels of PM2.5 exposure. In this study, stronger associations between long-term PM2.5 exposure and MI and overall mortality was observed as compared with stroke. It is stated that the PM2.5 levels observed in the study reflect average ambient exposure levels observed in the United States between 1988 and 2008; however, higher or lower average ambient PM2.5 levels may exist in the other geographic regions and the study findings may not be generalizable to populations exposed to higher levels of long-term ambient PM2.5 exposure. The study used a sophisticated spatiotemporal exposure model to estimate residential ambient PM2.5 levels biennially throughout the follow-up period, however, the information on time-activity patterns or personal PM2.5 exposures are not known. Participants reported the average duration and intensity of weekly physical activity, though the information on the time, location, variability, or duration of each activity and precise information on PM2.5 exposures specifically during physical activity is not known. Thus, the authors agree that there might be some measurement error in the PM2.5 estimates which make it more challenging to detect interaction between PM2.5 exposure and physical activity.

Hankey, et al. investigated the built environment’s association with air pollution and physical inactivity and estimated attributable health risks for policy implications. The study specifies that the results are relevant to health officials, sustainability scientists, and urban planners as it compares health risks for both air pollution and physical inactivity among neighbourhoods based on the activity patterns of residents in an urban area. It was found that attributes of the built environment were associated with both air pollution exposure and physical inactivity. These results emphasize how, in order to be health protective, neighbourhoods designed to decrease risks from one factor must avoid unintentionally increasing risks from other factors. Efforts to design healthy neighbourhoods should account for many factors, including air pollution and physical inactivity, and not address one concern at the expense of others.

Pollutants such as PM2.5, nitrogen oxides (NO_x_), and ozone (O_3_) were considered in the study and within-urban variability in physical inactivity and home-based air pollution exposure were estimated, followed by the estimation of risk for IHD. IHD mortality risks among neighbourhoods were estimated based on the ‘walkability’ scores. The results of the study suggest that differences in population health impacts on neighbourhoods are similar in magnitude for air pollution and physical activity. So, both physical activity and exposure to air pollution were found to be the critical aspects of planning for cleaner, health-promoting cities.

The study found only a small proportion of the population was physically active, however, the proportion of physically active individuals was higher in high- versus low-walkability neighbourhoods. Between-neighbourhood variability in estimated IHD mortality attributable to physical inactivity was less, at 7 fewer IHD deaths/100,000/year in high- vs. low-walkability neighbourhoods. Between-neighbourhood differences in estimated IHD mortality from air pollution were comparable in magnitude, at 9 more IHD deaths/100,000/year for PM2.5 and 3 fewer IHD deaths for O_3_ in high- vs. low-walkability neighbourhoods, suggesting that population health benefits from increased physical activity in high-walkability neighbourhoods may be offset by adverse effects of air pollution exposure.

The study was undertaken with the goal of understanding and designing clean, healthy, sustainable cities. It proposes further analyses incorporating other risk factors such as noise, transport injuries linked to the built environment; future analyses using age-specific risks of IHD mortality for air pollution and physical inactivity. More research is needed to explore causality between urban form and health risks, especially for physical activity, because ambient air pollution exposure is largely determined by geographical location.

A study by Lin et al., showed that active commuters over 45 years old had more CVD-free years and life expectancy than non-active commuters under lower PM2.5 concentration. However, the study pointed out that the beneficial effects of active commuting were counteracted by long-term exposure to high PM2.5 exposure. Significant multiplicative interaction of commuting mode and PM2.5 level was reflected in all-cause mortality, with the lowest risk observed in cycling participants exposed to a lower level of PM2.5. 

The study concluded that active commuting was associated with lower risk of CVD, all-cause mortality, and longer life expectancy among Chinese adults under ambient settings with lower PM2.5 level. Thus, it was found that active commuting among adults should be encouraged along with developing stringent strategies on reducing ambient PM2.5 for prevention of CVD and prolongation of life expectancy. This provided population-based evidence which is critical for policy making on environment protection and healthy lifestyle for people in developing countries with hazardous air pollution levels (especially PM2.5). 

The information of commuting behaviour and physical activity in the study were self-reported instead of objective measurements using pedometer etc. Thus, recall bias may introduce misclassification of commuting modes and attenuate the association of active commuting with outcomes. The information regarding commuting time and distance was not included in the analysis. Only data of ambient air PM2.5 were taken into consideration in the study; the health impacts of other ambient air pollutants and the effects of work-related or indoor air pollution were not taken into account in the study. Then, access to health care may not be the same for all participants which could have led to differential outcome misclassification. The study recommended follow-up surveys to obtain detailed information regarding the commuting modes and air pollution levels for more accurate estimates of the associations. 

Another study by Liu et al. [29] examined the impact of several commuting methods (walking, taking the subway, driving a car, and taking the bus) on heart rate variability (HRV) indices in young adults who were exposed to PM2.5 in urban regions with considerable traffic. The author discovered that people who were directly exposed to air pollution, such as those who walked or took the subway, had lower HRV indices. The authors could not control the confounding effects of respiration on HRV indices since the individuals’ breathing patterns may have affected ventricular outflow and they did not analyse other pollutants such carbon monoxide and nitrogen dioxide that may have confounded the effects. However, the link between PM2.5 and CVDs was highlighted by this study as it provides a solid foundation for reconsideration of, and further investigation into, the question of whether engaging in outdoor physical activity is beneficial or not when done in areas with high levels of PM2.5.

Another study [28] that examined the effects of O_3_ and NO_2_ exposure on LAP during physically active adults’ entire daily routine while exercising in both outdoor and indoor environment discovered that despite the higher O_3_ concentration exposure, physically active individuals may have a lower risk of developing cardiovascular and metabolic diseases, and the exposure while exercising did not pose an additional health risk. Lower triglyceride levels and lower LDL levels were seen in the outdoor group exposed to greater O_3_ levels compared to the untrained group. However, the study did not take into account PM2.5, which, together with O_3_ and NO_2_, is one of the three air pollutants having the greatest adverse effects on the health of people. In addition, the study did not distinguish the air pollutants with respect to specific activities. 

According to a study conducted on the Danish Diet, Cancer, and Health cohort by Andersen, et al. [23], the benefits of outdoor physical activity were not diminished by exposure to NO_2_; long-term effects of NO_2_ and physical activity on overall and cardiovascular mortality are independent of one another. The study comprised a sizable cohort of older adults (aged 50–65 years), and the level of physical activity among them was self-reported, based on their participation in sports, cycling, walking, and gardening. The NO_2_ levels near the participants’ residence were utilized by the authors as a stand-in for the typical air pollution levels that people experience while exercising. This premise holds true for gardening, which is frequently done at home but less so for cycling and walking. So, it is possible that the NOs in those who were cycling and walking were misclassified, which would have affected the study’s findings. Similarly, engaging in sports is a poor substitute for outdoor activity since we lack information on the nature of the sport or whether it was played indoors or outside. As a result, exposure misclassification may be to blame for the lack of results of a relationship between air pollution and sports participation. However, the study took into account each participant’s exposure to air pollution while evaluating the advantages of physical exercise at the individual level in an urban cohort. The effect of PM on CV mortality when engaging in outdoor physical exercise could not be confirmed since the study did not assess the PM levels that are probably harmful to health outcomes. Despite the study’s limitations, the strong interaction effects suggested that physical activity does have a good impact on cardiovascular health and may therefore be helpful in preserving CV health even when exposed to air pollution. In environments where PM levels may be high and the effect may change, it is necessary to exercise caution.

The protective effects of outdoor physical exercise on incidence and recurrent MI were demonstrated in a further cohort study [26] from the same DANISH cohort. However, the findings need to be interpreted with caution because the study had the same advantages and disadvantages as those mentioned previously.

Empirical research has highlighted the modifying role of PA on the effect of air pollution exposure on CVD outcomes, CVD health [32,33,34] and on respiratory health [35]. A high level of PA was found to be associated with stronger relationships between air pollutants and high blood pressure (SBP and DBP), and mean arterial pressure, as compared to the low levels of PA. Various studies have emphasized the risks involved in the exposure to the high concentration of ambient air pollutants during outdoor exercise as breathing rate and intensity increase leading to an increased inhaled quantity and deposition of air pollutants in the body [35,36]. Therefore, there is evidence available for both, the health gains from PA and the concerns regarding the potential health risks from increased air pollution exposure [7].

None of the seven studies included in the current systematic review evaluated the effect on CVS in a comprehensive manner; two studies investigated mortality due to diabetes [23] and total (all-cause) mortality [23,24] apart from some cardiovascular indicators. Although studying the effect on the respiratory system was beyond the scope of the current systematic review, it is important to mention here that there are many studies and systematic reviews that have evaluated the effect of pollutants on the respiratory system, however, there is relatively less evidence in the context of CVDs. This may be because the effect on the respiratory system is more than that on the cardiovascular system and/or the indicators for evaluating respiratory health are less sensitive and more reliable than the indicators used for CVDs.

Most of the studies indicate that the adverse effects of PA in an outdoor polluted environment effect the respiratory system more than they do the cardiovascular system [37,38,39]. This is likely because moderate exercise has a direct positive effect on cardiovascular health as it improves different CV indices and markers, and reduces the incidence of MIs, angina, etc. While air pollution directly effects the respiratory system, i.e., the worsening of various respiratory indices and markers, and exercising in a polluted environment has a direct negative impact on the respiratory system, therefore, the beneficial effects of PA are outweighed by the harmful effects of air pollution.

A scoping review by Sun and Zhu in 2019 reported that respiratory and CVD mortality resulting from outdoor air pollution exposure were the most common health outcomes after the overall mortality—when the broad research areas in this context were reviewed [39]. A systematic review and meta-analysis completed by Qin et al., 2019 [38] on exercise and air pollutant exposure stated that six studies (out of the total 25 studies included in the review) demonstrated that exposure to traffic pollution while exercising may contribute to changes in blood pressure, systemic conduit artery function and microvascular function. The combined effect of air pollution and exercise was found to be associated with an increased risk of cardiopulmonary health and immunity. Also, cardiovascular health seems to be less affected as compared to respiratory health when each of these are considered in isolation. It is important to note that this systematic review [38] included studies with an intervention or a prescribed dosage of exercise while our systematic review excluded experimental designs.

Some of the studies in our systematic review were completed with the aim of understanding and reviewing policy implications for city planning assuming or observing that higher walkability areas lead to more PA among the population (2 out of 7) living in an area. So, in this context measuring air pollution exposure and modes of transportation becomes an important consideration. In one of the studies [25], the main aim was to supplement the policy implications and city planning to increase PA through better neighbourhood design. The study interpreted that the differences in the impact of air pollution and PA on population health among neighbourhoods are similar in magnitude, so, PA and air pollution exposure are critical aspects of planning for health-promoting cities. However, it is important to note that the main objective of the study was to investigate the built environment (in terms of walkability) and the impact of air pollution and physical inactivity on the estimated IHD mortality.

Another study [29] included in our systematic review also iterated the implications for public health policy and recommended subways or rapid public transit system in urban areas for both total air quality improvement and commuter health improvement. The study recommended that people should be encouraged to use rapid public transit systems instead of walking during rush hours as the study found that exposure to air pollutants was significantly higher among commuters who walked as compared with people who used subways. Interestingly, the study highlighted how AC usage and circulation within the vehicles (including frequent door opening and closing, efficiency of the AC system) is an important factor to be considered as far as the concentration of air pollutants in the vehicle is concerned; commuters in gas-powered buses with an AC system had a lesser air pollution exposure and HRV decrement as compared with commuters who walked.

On the other hand, a systematic review on ‘Built environment and health’ by Schulz, Romppel and Grand in 2018 highlighted how evidence indicates that higher levels of noise and air pollution are related to higher blood pressure levels while no association was observed between green spaces or street design and health [40]. The review concluded that future research should investigate the complex relationship between the built environment and health using a sound theoretical basis and better research designs.

A study undertaken by Cunningham, 2020 [41] advocated for the provision of PA opportunities to improve the health of populations as it offsets the negative effects of air pollution. Since the behaviour of engaging oneself in PA is modifiable as compared to some of the causes of pollution, providing population with better access to PA can be an effective policy tool for improving population health. The study points out that residents were less likely to report poor health, and fewer physically unhealthy days in the countries where access to PA were plentiful.

Another review article by Giles 2014 [37], pointed out that, apart from the effect on respiratory and cardiovascular systems and other systemic effects, air pollution exposure during exercise affects maximal oxygen consumption and exercise performance. The review article highlighted the elevated risks (elevated heart rate, myocardial ischemia, impairment of lung function and increased lung inflammation) involved with respect to populations with compromised cardiovascular and/or respiratory function with exposure to acute and chronic PM2.5, carbon monoxide or ozone prior to exercise. This review emphasized the need to avoid exposure to air pollution while engaging in PA and people should be encouraged to exercise away from traffic. The importance of elevated ambient temperature was also highlighted in the article as it increases perceived exertion and air pollution-induced lung inflammation. The article gave recommendations such as: avoiding time period/s of maximum air pollution levels; following local air quality forecasts and planning workouts around them; and avoiding exercise by at-risk individuals (pre-existing cardiovascular or respiratory disease or other serious disorders) until further research has been conducted and we know more about the effects of exercising in an air-polluted environment. The importance of the right environment, land-use patterns, selection of exercise locations and mode of transportation to an exercise location were highlighted as ‘integral’, especially in an urban context. The article pointed towards the need for scientific research to explore the role of PM exposure during exercise on cognition and systemic inflammation given the link between PM exposure and impaired vascular function.

However, another review by Gladwell et al. [42] highlighted the importance of engaging in PA in an outdoor environment. The authors in the review pointed out the synergistic effect of exercise and exposure to a nature/outdoor environment can be an important strategy to help fight the rising incidence of non-communicable diseases in the population. Thus, it is important to strike a balance between PA and exposure to outdoor environments, especially in the urban context. Another non-systematic mapping review of empirical and modelling evidence of the possible links between exposure to air pollutants and physical activity by Tainio et al. [43] shows that observational epidemiological studies have found small diminishing health gains from PA in an air polluted environment for long-term outcomes. Further, the review argues that the public health modelling studies have estimated that the benefits of PA outweigh the risk of air pollution particularly in the active transport environment.

While we discuss the positive and negative results of exercising outdoors in a polluted environment, it becomes important to understand the modulating effects of a healthy lifestyle that includes dietary habits and behaviours (health or unhealthy). One of the studies in this systematic review reports a positive effect of exercise on cardiovascular and metabolic diseases irrespective of the higher O_3_ concentration exposure during exercise. The study argues that lower BMI and HR among the exercising group (indoor or outdoor) could be due to healthy eating behaviour and lifestyle among them. This shows that those who are active and regularly engage in exercise (outdoor or indoor), benefit from a healthy lifestyle and healthy eating habits. Several studies have highlighted the beneficial effects of a healthy lifestyle, including healthy dietary habits, on cardiovascular health [28]. Therefore, it is important that people should adopt healthy dietary habits for improved cardiovascular health as this would aid in balancing or reversing the adverse effects of ambient air pollution.

### Strengths and Limitations of the Review

This is a unique systematic review which investigated the interaction between engaging in PA in an air polluted environment and CVD risk. Most of the recent reviews [43,44] in this area are non-systematic in nature and did not distinguish between outdoor and indoor PA. Therefore, the effect of performing PA in an outdoor polluted environment on cardiovascular health could not be established. The present review, therefore, sought to have a broader perspective regarding the impact on cardiovascular health among people engaging in an outdoor PA. This indirectly gave us an opportunity to study the impact of air pollution on the general population at large as no experimental studies were included in the given systematic review.

Only observational studies were included in the current systematic review as the review’s objective was to measure and understand the prevalence and/or incidence of cardiovascular outcomes and its probable association when people engage in outdoor PA in a polluted environment. Studies included in the systematic review were conducted in developed countries, therefore, their generalizability for developing countries is limited. Hence, more studies are needed in developing countries. In general, the experimental studies consider limited exposure categories which are specific in nature, so, this systematic review focused on reviewing a broader array of exposures and types of PAs. Experimental studies have their own advantages; however, they often restrict or limit the generalization of results.

Further, restricting the outcomes to the cardiovascular system helped to understand the joint effects of outdoor exercise and polluted environment on cardiovascular health per se; to study the prevalence and incidence of cardiovascular morbidities and mortality in the general population engaging in outdoor exercise.

The methods adopted for the measurement of levels of various pollutants varied from study to study, and the same was observed in the context of PA as well. Some studies used self-administered questionnaires while others used WHO MET Guidelines and other studies used instruments such as Actigraph to measure PA. The outcomes also varied across the studies and the data were heterogeneous to be combined in a meta-analysis. Quantitative pooling was not possible in the present systematic review because of the substantial heterogeneity and methodological limitations of the original studies. Thus, a narrative synthesis of the included studies has been presented in this systematic review. It is important to note here that most of the included studies had limitations in terms of sample size, methods of pollutants and PA assessment, and lack of adjustment for potential confounders.

A methodological challenge that likely contributes to the inconsistent evidence is that both air pollution and PA may have short and long-term effects on the vascular system of the body. None of the studies took this into account. The segregation of short and long-term effect of air pollution and PA was not considered. Moreover, the studies were mostly done with adult or older age groups (age group > 19 years of age), therefore, the results cannot be generalized for younger populations.

Further, none of the studies included pollutants such as carbon monoxide, SO_2_, TVOCs, dioxins, and Polycyclic Aromatic Hydrocarbons (PAHs) and the combined effects of all the air pollutants on the cardiovascular system. This systematic review did not consider the effects of exercising in a polluted environment on the cardio-pulmonary system or on the overall health of an individual or population at large.

## 5. Conclusions

The findings of the present systematic review indicate that the beneficial effects of outdoor PA outweigh the harmful effects of air pollution on cardiovascular health, however, the benefits are reduced when the exercise/PA is performed in an outdoor polluted environment. PM2.5 seems to be an important pollutant that might alter the effect of the PA on cardiovascular health. Therefore, it is imperative to consider the levels or concentration of major individual pollutant/s, such as PM2.5, NO_x_, SO_x_, O_3_ etc., on cardiovascular outcomes when people are engaged in outdoor physical activity. This systematic review showed the varied effects of different pollutants, although the negative effect of PM2.5 was found to be higher as compared with other pollutants.

All the studies included in the systematic review recommended large prospective studies to further explore the relation between engagement in PA in an outdoor polluted environment and cardiovascular health. The studies also reinforced the need for investment in this area of research. It should be noted that most of the studies in the systematic review have been done in developed countries, so, there is an urgent need to undertake similar primary studies in developing countries to understand the effects of performing outdoor physical activity in a polluted environment in these countries. Age stratification was different in studies included in the systematic review. Future research should be undertaken for the younger population, especially looking into the long-term effects of engaging in PA in a polluted environment (considering all the harmful pollutants including PM2.5, NO_2_ and O_3_) on health per se.

Further, only seven studies were eligible for inclusion in this review, due to the lack of distinction between outdoor and indoor PA and its effects on cardiovascular health. We recommend that: (1) more studies with robust and uniform methodology should be done, especially considering PM2.5; (2) future studies should include pollutants such as carbon monoFxide, SOX, TVOCs, dioxins, polycyclic aromatic hydrocarbons (PAHs) and the combined effects of all the air pollutants on cardiovascular health; (3) studies should be undertaken with adequate sample size and measures to control and adjust the potential confounders; (4) future research studies should clearly differentiate between outdoor and indoor physical activity; (5) adapting uniform assessment criteria or measurement protocols to quantify the exposure and outcomes; (6) developing countries to undertake more studies in this direction; (7) the changes or variations in the systemic responses to exercise-linked pollutant exposure, including temperature, exercise intensity and sex in different age-cohorts such as the elderly, young, and those with pre-existing diseases should be studied. Healthy lifestyle including healthy dietary habits and behaviours shape a healthy cardiovascular profile of individuals. People should follow a healthy lifestyle to counter the harmful effects of air pollution, and studies are warranted in this area as well.

## Figures and Tables

**Table 1:** Characteristics of the included studies (*n* = 7).

Author & Year	Objective/s of the Study	Study Design	Settings/Country	Period	Sample Size	Age (Years)	Physical Activity	Pollutants
Andersen 2015 [23]	To examine whether the effect of PA in the outdoor environment on mortality is moderated by long-term exposure to high air pollution levels.	Used data from a prospective urban cohort from Danish Diet, Cancer, and Health cohort.	People living in Aarhus and Copenhagen, Denmark	1993–1997 followed till 2010	52,061	50–65 years	PA was reported as hours per week spent on sports, cycling, gardening, walking, housework and “do-it-yourself” activities.	NO_2_
Elliott, et al., 2020 [24]	To examine the multiplicative interaction between long-term ambient residential exposure to fine PM2.5 and PA in association with CVD risk and overall mortality.	Nationwide prospective Cohort study	United States; NHS participant’s self-administered questionnaires biennially, providing information on incident diseases, medical history, and lifestyle factors, along with leisure-time PA.	NHS participants from 1988 to 2008	104,990 eligible participants	30–55 year old females	Leisure-time PA was collected using information from the biennial questionnaires.	PM2.5; Monthly average PM2.5 and/or PM10 monitoring data from the U.S. Environmental Protection Agency’s Air Quality
Hankey, et al., 2012 [25]	Study investigated the built environment’s association with air pollution and physical inactivity, and estimated attributable health risks	Cross-sectional study	Six counties of Southern California, United States; 2001 Post-Census Regional travel survey data were used to estimate within-urban variability in physical inactivity and home-based air pollution exposure.	During fall 2001 and spring 2002.	30,007	21–54 years	Study used 2001 Post- Census Regional Travel Survey; A geocoded time-activity diary was used to capture self-reported activities and travel.	PM2.5, NO_x_, and O_3_
Kubesch 2018 [26]	To determine the effects of leisure-time and transport-related PAs on the risk of incident and recurrent MI in middle-aged men and women, and to examine whether these effects were modified by residential exposure to TRAP.	Retrospective Cohort Study	Data from the Danish Diet, Cancer, and Health cohort, living in Copenhagen or Aarhus, Denmark	1993–1997; MI, reported until December 2015	51,868	50–64 years	Self-administered, interviewer-checked questionnaire was used, which included leisure-time and utilitarian physical activities were reported as hours per week (hours/week) spent on sports and “do-it-yourself” activities.	NO_2_
Lin (2021) [27]	To investigate the association of commuting mode with CVD incidence, mortality, and life expectancy, and to further evaluate the counteractive effect of long-term ambient PM2.5 on such associations, using large cohorts of general Chinese population from China-PAR.	Prospective cohort study of general Chinese population and the data were from three cohorts of China-PAR project	China; Participants were from three cohorts of the China-PAR project, including China MUCA (1998), InterASIA and CIMIC.	China MUCA (1998), InterASIA and CIMIC were established in 1998, 2000–2001 and 2007–2008, respectively. China MUCA (1998) and InterASIA were followed up twice during 2007–2008 and 2012–2015, and CIMIC was followed up once during 2012–2015	76,176	51.2 ± 11.8 (Mean ± SD)	PA was assessed based on the mode of commuting. Questions were asked on commuting to and from work and the op- tions were “A. Walking; B. Cycling; C. Public transportation; D. Driving (Car or motorcycle); E. No need to commute (i.e., working at home)”. Commuting modes were categorized into three types: non-active (including public transportation, driving, and no need to commute), walking and cycling. Non-active commuting mode was served as reference in the study.	Ambient PM2.5 was assesed. Satellite-based spatiotemporal models were applied to estimate environmental fine particulate matter (PM2.5) exposure levels. monthly PM2.5 concentration in China from 2000 to 2015. The resi- dential address of each participant was geocoded as a grid cell according to latitude and longitude data. The average monthly PM2.5 concentra- tion from 2007 to 2008 survey to 2015 were calculated for each participant.
Marmett (2022) [28]	To evaluate the effects of O_3_ and NO_2_ exposure on cardiorespiratory fitness, LAP, and environmental health risk during the entire daily routine of physically active adults that exercise outdoor and indoor environments.	Cross-sectional study.	Brazil; The participants included in the study were from local universities of the Metropolitan Region of Porto Alegre.	November 2018 to February 2020	One-hundred twenty healthy young men assigned to three groups: untrained (*n* = 52), indoor (*n* = 36), and outdoor exercise (*n* = 32).	18–45 years		O_3_ and NO_2_
Wen-Te Liu 2015 [29]	To investigate the built environment’s association with air pollution and physical inactivity, and estimated attributable health risks.	Panel Study	Taiwan; healthy students from universities in Taipei, Taiwan, commuting via electrically powered subway, a gas-powered bus, a gasoline-powered car, and walking.	2012–2014	120	19–24 years	Four different commuting modes—an electrically powered subway, a gas-powered bus, a gasoline-powered car, and walking for each student during 1-h morning commutes was considered.	PM10 and PM2.5

Abbreviations: China MUCA: China Multi-Center Collaborative Study of Cardiovascular Epidemiology; China-PAR: Prediction for Atherosclerotic Cardiovascular Disease Risk in China project.; CIMIC: Community Intervention of Metabolic Syndrome in China and Chinese Family Health Study; CVD: Cardio Vascular Diseases; InterASIA: International Collaborative Study of Cardiovascular Disease in Asia; LAP: lipid accumulation product; MI: Myocardial Infarction; NHS: Nurses’ Health Study; NO_2_: nitrogen dioxide; NO_x_: nitrogen oxides; O_3_: ozone; PA: physical activity; PM: particulate matter; SD: standard deviation; TRAP: traffic related air pollution.

**Table 2:** Measurement of independent variables.

Study	Measurement of Physical Activity	Measurement of Pollutants/Pollution
Andersen 2015 [23]	PA was assessed by a self-administered, interviewer-checked questionnaire in which leisure time and transport-related PA were reported as hours per week spent on sports, cycling, gardening, walking, housework and “do-it-yourself” activities.	Mean of annual concentrations of NO_2_ at residential addresses of each cohort participant were used; since 1971 until the end of follow-up. This was used as a proxy of average exposure to TRAP during exercise. The authors defined an indicator variable of high versus moderate/low NO_2_ exposure separated by the 75th percentile of the exposure range in the cohort (≥ vs. <19.0 μg/m^3^).
Elliott, et al., 2020 [24]	Study measured leisure-time PA information from biennial questionnaires. Participants reported the average time per week they spent in specific leisure-time activities, and other low and high-intensity activities in seven provided categories, ranging from 0 min to ≥11 h/week. The time per week spent participating in each activity was multiplied by each activity’s metabolic equivalent of task (MET) score to obtain MET-hours per week, which incorporates frequency, duration, and intensity of activity. The overall physical activity in MET-hours per week by summing the MET-hours per week across all activities.	Residential addresses were updated every two years with each questionnaire cycle and geocoded to obtain latitude and longitude. Exposure to PM2.5 at each residential address using spatiotemporal prediction models was calculated. The generalized additive mixed models used monthly average PM2.5 and/or PM10 monitoring data from the U.S. Environmental Protection Agency’s Air Quality System and other publicly available networks. Models also incorporated geospatial predictors and monthly average meteorological. Moving averages for each 24-month questionnaire cycle as a measure of long-term exposures were calculated.
Hankey, et al., 2012 [25]	Study used 2001 Post- Census Regional Travel Survey to estimate within-urban variability in physical inactivity. A geocoded time-activity diary was used to capture self-reported activities and travel undertaken. The survey covered one weekday per participant and multiplied each participant’s one-day PA record by seven to obtain an estimate of weekly minutes of PA.	Study estimated the annual average of daily one-hour maximum concentrations for O_3_ and annual-average concentrations for PM2.5 and NO_x_ at each survey participant’s residence.
Kubesch 2018 [26]	In the Danish Diet, Cancer and Health cohort, information on physical activities was collected by a self-administered, interviewer-checked questionnaire in which leisure-time and utilitarian physical activities were reported as hours per week (h/week) spent on sports, cycling, gardening, walking, housework, and “do-it-yourself” activities. Information was collected separately for winter and summer of the previous year, and the 2 values were averaged, so that being active implies at least half an hour per week spent on a specific activity. Participants reported their level of physical activities at work at the time of enrolment defined as sedentary, standing, light, or heavy.	The annual mean concentrations of NO_2_ at residential addresses of each cohort participant as a proxy of average exposure to traffic-related air pollution in general, and during exercise. The mean NO_2_ concentrations correspond to each participant’s recruitment year and therewith to the same year for which participants reported physical activities. The indicator of low (lower 25th percentile of exposure range: <14.3 ug/m^3^), medium (≥14.3–21.0 ug/m^3^), and high (upper 25th percentile of exposure range: ≥21.0 ug/m^3^) exposure to NO_2_.
Lin (2021) [27]	PA was assessed based on the mode of commuting. Questions were asked on commuting to and from work and the options were “A. Walking; B. Cycling; C. Public transportation; D. Driving (Car or motorcycle); E. No need to commute (i.e., Working at home)”. Commuting modes were categorized into three types: non-active (including public transportation, driving, and no need to commute); walking; and cycling. The non-active commuting mode was served as reference in the study.	Ambient PM2.5 was assessed. Satellite-based spatiotemporal models were applied to estimate environmental fine particulate matter (PM2.5) exposure levels and monthly PM2.5 concentration in China from 2000 to 2015. The residential address of each participant was geocoded as grid cells according to latitude and longitude data. The average monthly PM2.5 concentration from 2007 to 2008 survey to 2015 were calculated for each participant.
Marmett (2022) [28]	Participants in the study engaged in physical training programs (>150 min/week) for six months before the experimental trial for exercised groups or remained untrained for six months before the experimental trial for the untrained group (<2 exercise sessions/week). The classification criteria for trained and untrained individuals were based on the ‘Physical Activity Guidelines Advisory Committee Scientific Report’ (2018), and for time of exercise of >150 min/week based on the WHO recommendation of physical activity for adults, which indicates 150–300 min of moderate-intensity aerobic physical activity. PA included the frequency and duration of activities as walking, moderate and vigorous intensities. The product of PA intensity (MET) and duration (h) was calculated as the volume of the activity (MET-h)	Personal exposure samplers of O_3_ and NO_2_ were used to measure the concentration of the pollutants through passive monitoring. All participants received two personal exposure samplers to monitor O_3_ and NO_2_ for a period of 24 h and seven days, respectively. The concentrations of O_3_ and NO_2_ were expressed as lg/m 3/8 h and lg/m 3/24 h, respectively
Wen-Te Liu 2015 [29]	Four different commuting modes were considered—an electrically powered subway, a gas-powered bus, a gasoline-powered car, and walking. Students underwent three one-hour measurements (each measurement for each ride for a total of three rides per participant), resulting in a total of 360 one-hour measurements between January and March in the years 2012 to 2014. Students’ age, sex, body mass index (BMI) and time-activity pattern information were recorded with a questionnaire during their measurements.	One-hour continuous air pollution monitoring during each measurement for each participant. PM < 10 um in aerodynamic diameter (PM10), PM < 2.5 m in aerodynamic diameter (PM2.5), temperature, and relative humidity were measured continuously using a personal dust monitor which measured and recorded one-minute mass concentrations of PM10 and PM2.5 and temperature and relative humidity. Total volatile organic compounds (TVOCs) were measured continuously using a TVOCs monitor.

## Data Availability

Not applicable.

## Supplementary Materials

The following supporting information can be downloaded at: https://www.mdpi.com/article/10.3390/ijerph191710547/s1, Table S1: Search strings from PubMed, Web of Science, and Embase; Table S2: Quality Appraisal Checklist for Cohort Studies and Cross-Sectional Studies; Table S3: Summary of Findings. Reference [45] are cited in the supplementary materials.
