# Supplementary material for: Outdoor Physical Activity in an Air Polluted Environment and Its Effect on the Cardiovascular System—A Systematic Review
Source: Int J Environ Res Public Health. 2022 Aug 24;19(17):10547. doi: 10.3390/ijerph191710547 (PMC9517891; doi:10.3390/ijerph191710547)
Supplement: Supplementary file 1 [file ijerph-19-10547-s001.zip › ijerph-1744376-supplementary.pdf]

Supplementary Files

Search Strings:

Table S1.a: Search string from PubMed:

Search number	Query	Filters	Search Details
12	((#7) AND (#8)) AND (#9)	English, Humans	(("air pollution"[MeSH Terms] OR "air pollutants"[MeSH Terms] OR "soot"[MeSH Terms] OR "carbon monoxide"[MeSH Terms] OR "lead"[MeSH Terms] OR "nitrogen dioxide"[MeSH Terms] OR "nitrogen oxides"[MeSH Terms] OR "ozone"[MeSH Terms] OR "particulate matter"[MeSH Terms] OR "sulfur dioxide"[MeSH Terms] OR "sulfur oxides"[MeSH Terms] OR ("ambient air pollut*"[Title/Abstract] OR "outdoor air pollut*"[Title/Abstract] OR "PM10"[Title/Abstract] OR "PM25"[Title/Abstract])) AND ("exercise"[MeSH Terms] OR "exercise therapy"[MeSH Terms] OR "games, recreational"[MeSH Terms] OR "jogging"[MeSH Terms] OR "leisure activities"[MeSH Terms] OR "running"[MeSH Terms] OR "sports"[MeSH Terms] OR "walking"[MeSH Terms] OR ("cycl*"[Title/Abstract] OR "outdoor physical activi*"[Title/Abstract] OR "physical activit*"[Title/Abstract])) AND ("cardiovascular diseases"[MeSH Terms] OR "cardiovascular system"[MeSH Terms] OR "heart diseases"[MeSH Terms] OR "morbidity"[MeSH Terms] OR "mortality"[MeSH Terms] OR "vascular diseases"[MeSH Terms] OR ("adverse effect*"[Title/Abstract] OR "cardiac event*"[Title/Abstract] OR "cardiac outcome*"[Title/Abstract] OR "cardiovascular disorder*"[Title/Abstract] OR "cardiovascular event*"[Title/Abstract] OR "cardiovascular outcome*"[Title/Abstract] OR "circulatory system"[Title/Abstract] OR "CVD"[Title/Abstract] OR "vascular event*"[Title/Abstract] OR "vascular outcome*"[Title/Abstract]))) AND ((humans[Filter]) AND (english[Filter]))

11	((#7) AND (#8)) AND (#9)	Eng l ish	(("air pollution"[MeSH Terms] OR "air pollutants"[MeSH Terms] OR "soot"[MeSH Terms] OR "carbon monoxide"[MeSH Terms] OR "lead"[MeSH Terms] OR "nitrogen dioxide"[MeSH Terms] OR "nitrogen oxides"[MeSH Terms] OR "ozone"[MeSH Terms] OR "particulate matter"[MeSH Terms] OR "sulfur dioxide"[MeSH Terms] OR "sulfur oxides"[MeSH Terms] OR ("ambient air pollut*"[Title/Abstract] OR "outdoor air pollut*"[Title/Abstract] OR "PM10"[Title/Abstract] OR "PM25"[Title/Abstract])) AND ("exercise"[MeSH Terms] OR "exercise therapy"[MeSH Terms] OR "games, recreational"[MeSH Terms] OR "jogging"[MeSH Terms] OR "leisure activities"[MeSH Terms] OR "running"[MeSH Terms] OR "sports"[MeSH Terms] OR "walking"[MeSH Terms] OR ("cycl*"[Title/Abstract] OR "outdoor physical activi*"[Title/Abstract] OR "physical activit*"[Title/Abstract])) AND ("cardiovascular diseases"[MeSH Terms] OR "cardiovascular system"[MeSH Terms] OR "heart diseases"[MeSH Terms] OR "morbidity"[MeSH Terms] OR "mortality"[MeSH Terms] OR "vascular diseases"[MeSH Terms] OR ("adverse effect*"[Title/Abstract] OR "cardiac event*"[Title/Abstract] OR "cardiac outcome*"[Title/Abstract] OR "cardiovascular disorder*"[Title/Abstract] OR "cardiovascular event*"[Title/Abstract] OR "cardiovascular outcome*"[Title/Abstract] OR "circulatory system"[Title/Abstract] OR "CVD"[Title/Abstract] OR "vascular event*"[Title/Abstract] OR "vascular outcome*"[Title/Abstract]))) AND (english[Filter])
10	((#7) AND (#8)) AND (#9)	-	("air pollution"[MeSH Terms] OR "air pollutants"[MeSH Terms] OR "soot"[MeSH Terms] OR "carbon monoxide"[MeSH Terms] OR "lead"[MeSH Terms] OR "nitrogen dioxide"[MeSH Terms] OR "nitrogen oxides"[MeSH Terms] OR "ozone"[MeSH Terms] OR "particulate matter"[MeSH Terms] OR "sulfur dioxide"[MeSH Terms] OR "sulfur

			oxides"[MeSH Terms] OR ("ambient air pollut*"[Title/Abstract] OR "outdoor air pollut*"[Title/Abstract] OR "PM10"[Title/Abstract] OR "PM25"[Title/Abstract])) AND ("exercise"[MeSH Terms] OR "exercise therapy"[MeSH Terms] OR "games, recreational"[MeSH Terms] OR "jogging"[MeSH Terms] OR "leisure activities"[MeSH Terms] OR "running"[MeSH Terms] OR "sports"[MeSH Terms] OR "walking"[MeSH Terms] OR ("cycl*"[Title/Abstract] OR "outdoor physical activi*"[Title/Abstract] OR "physical activit*"[Title/Abstract])) AND ("cardiovascular diseases"[MeSH Terms] OR "cardiovascular system"[MeSH Terms] OR "heart diseases"[MeSH Terms] OR "morbidity"[MeSH Terms] OR "mortality"[MeSH Terms] OR "vascular diseases"[MeSH Terms] OR ("adverse effect*"[Title/Abstract] OR "cardiac event*"[Title/Abstract] OR "cardiac outcome*"[Title/Abstract] OR "cardiovascular disorder*"[Title/Abstract] OR "cardiovascular event*"[Title/Abstract] OR "cardiovascular outcome*"[Title/Abstract] OR "circulatory system"[Title/Abstract] OR "CVD"[Title/Abstract] OR "vascular event*"[Title/Abstract] OR "vascular outcome*"[Title/Abstract]))
9	(#5) OR (#6)	-	"cardiovascular diseases"[MeSH Terms] OR "cardiovascular system"[MeSH Terms] OR "heart diseases"[MeSH Terms] OR "morbidity"[MeSH Terms] OR "mortality"[MeSH Terms] OR "vascular diseases"[MeSH Terms] OR "adverse effect*"[Title/Abstract] OR "cardiac event*"[Title/Abstract] OR "cardiac outcome*"[Title/Abstract] OR "cardiovascular disorder*"[Title/Abstract] OR "cardiovascular event*"[Title/Abstract] OR "cardiovascular outcome*"[Title/Abstract] OR "circulatory system"[Title/Abstract] OR "CVD"[Title/Abstract] OR "vascular event*"[Title/Abstract] OR "vascular outcome*"[Title/Abstract]
8	(#3) OR (#4)	-	"exercise"[MeSH Terms] OR "exercise therapy"[MeSH Terms] OR "games,

			recreational"[MeSH Terms] OR "jogging"[MeSH Terms] OR "leisure activities"[MeSH Terms] OR "running"[MeSH Terms] OR "sports"[MeSH Terms] OR "walking"[MeSH Terms] OR "cycl*"[Title/Abstract] OR "outdoor physical activi*"[Title/Abstract] OR "physical activit*"[Title/Abstract]
7	(#1) OR (#2)	-	"air pollution"[MeSH Terms] OR "air pollutants"[MeSH Terms] OR "soot"[MeSH Terms] OR "carbon monoxide"[MeSH Terms] OR "lead"[MeSH Terms] OR "nitrogen dioxide"[MeSH Terms] OR "nitrogen oxides"[MeSH Terms] OR "ozone"[MeSH Terms] OR "particulate matter"[MeSH Terms] OR "sulfur dioxide"[MeSH Terms] OR "sulfur oxides"[MeSH Terms] OR "ambient air pollut*"[Title/Abstract] OR "outdoor air pollut*"[Title/Abstract] OR "PM10"[Title/Abstract] OR "PM25"[Title/Abstract]
6	((((((((adverse effect*[Title/Abstract]) OR (cardiac event*[Title/Abstract])) OR (cardiac outcome*[Title/Abstract])) OR (cardiovascular disorder*[Title/Abstract])) OR (cardiovascular event*[Title/Abstract])) OR (cardiovascular outcome*[Title/Abstract])) OR (circulatory system[Title/Abstract])) OR (CVD[Title/Abstract])) OR (vascular event*[Title/Abstract])) OR (vascular outcome*[Title/Abstract])	-	"adverse effect*"[Title/Abstract] OR "cardiac event*"[Title/Abstract] OR "cardiac outcome*"[Title/Abstract] OR "cardiovascular disorder*"[Title/Abstract] OR "cardiovascular event*"[Title/Abstract] OR "cardiovascular outcome*"[Title/Abstract] OR "circulatory system"[Title/Abstract] OR "CVD"[Title/Abstract] OR "vascular event*"[Title/Abstract] OR "vascular outcome*"[Title/Abstract]
5	(((((cardiovascular diseases[MeSH Terms]) OR (cardiovascular system[MeSH Terms])) OR (heart diseases[MeSH Terms])) OR (morbidity[MeSH Terms])) OR (mortality[MeSH Terms])) OR (vascular diseases[MeSH Terms])	-	"cardiovascular diseases"[MeSH Terms] OR "cardiovascular system"[MeSH Terms] OR "heart diseases"[MeSH Terms] OR "morbidity"[MeSH Terms] OR "mortality"[MeSH Terms] OR "vascular diseases"[MeSH Terms]
4	((Cycl*[Title/Abstract]) OR (outdoor physical activi*[Title/Abstract])) OR (physical activit*[Title/Abstract])	-	"cycl*"[Title/Abstract] OR "outdoor physical activi*"[Title/Abstract] OR "physical activit*"[Title/Abstract]

3	((((((exercises[MeSH Terms]) OR (Games, Recreational[MeSH Terms])) OR (jogging[MeSH Terms])) OR (leisure activities[MeSH Terms])) OR (Running[MeSH Terms])) OR	-	"exercise"[MeSH Terms] OR "exercise therapy"[MeSH Terms] OR "games, recreational"[MeSH Terms] OR "jogging"[MeSH Terms] OR "leisure activities"[MeSH Terms] OR
	(sports[MeSH Terms])) OR (walking[MeSH Terms])		"running"[MeSH Terms] OR "sports"[MeSH Terms] OR "walking"[MeSH Terms]
2	(((ambient air pollut*[Title/Abstract]) OR (outdoor air pollut*[Title/Abstract])) OR (PM10[Title/Abstract])) OR (PM2·5[Title/Abstract])	-	"ambient air pollut*"[Title/Abstract] OR "outdoor air pollut*"[Title/Abstract] OR "PM10"[Title/Abstract] OR "PM25"[Title/Abstract]
1	((((((((((air pollution[MeSH Terms]) OR (air pollutants[MeSH Terms])) OR (carbon black[MeSH Terms])) OR (carbon monoxide[MeSH Terms])) OR (lead[MeSH Terms])) OR (nitrogen dioxide[MeSH Terms])) OR (nitrogen oxides[MeSH Terms])) OR (ozone[MeSH Terms])) OR (particulate matter[MeSH Terms])) OR (sulfur dioxide[MeSH Terms])) OR (sulfur oxides[MeSH Terms])	-	"air pollution"[MeSH Terms] OR "air pollutants"[MeSH Terms] OR "soot"[MeSH Terms] OR "carbon monoxide"[MeSH Terms] OR "lead"[MeSH Terms] OR "nitrogen dioxide"[MeSH Terms] OR "nitrogen oxides"[MeSH Terms] OR "ozone"[MeSH Terms] OR "particulate matter"[MeSH Terms] OR "sulfur dioxide"[MeSH Terms] OR "sulfur oxides"[MeSH Terms]

Table S1.b: Search string from Web of Science:

#1	TS=("air pollutant*" OR "air pollution" OR "ambient air pollut*" OR "carbon black" OR "carbon monoxid*" OR "lead" OR "nitrogen dioxid*" OR "nitrogen oxid*" OR "outdoor air pollut*" OR "ozone" OR "particulate matter*" OR "PM10" OR "PM2·5" OR “sulfur dioxid*" OR "sulfur oxid*")<br>Indexes=SCI-EXPANDED, SSCI, A&HCI, ESCI Timespan=All years
#2	TS=(Cycl* OR exercis* OR Game* OR Recreation* OR Jogging OR "leisure activit*" OR "outdoor physical activi*" OR "physical activit*" OR Running OR sport* OR walking) Indexes=SCI-EXPANDED, SSCI, A&HCI, ESCI Timespan=All years
#3	TS=("adverse effect*" OR "cardiac event*" OR "cardiac outcome*" OR "cardiovascular diseas*" OR "cardiovascular disorder*" OR "cardiovascular event*" OR "cardiovascular outcome*" OR "cardiovascular system" OR "Circulatory system" OR CVD* OR "Heart Diseas*" OR morbidit* OR Mortalit* OR "Vascular Disease*" OR "vascular event*"OR "vascular outcome*")<br>Indexes=SCI-EXPANDED, SSCI, A&HCI, ESCI Timespan=All years
#4	#3 AND #2 AND #1<br>Indexes=SCI-EXPANDED, SSCI, A&HCI, ESCI Timespan=All years
#5	#3 AND #2 AND #1<br>Refined by: LANGUAGES: (ENGLISH)<br>Indexes=SCI-EXPANDED, SSCI, A&HCI, ESCI Timespan=All years
#6	#3 AND #2 AND #1<br>Refined by: LANGUAGES: (ENGLISH) AND DOCUMENT TYPES: (ARTICLE)<br>Indexes=SCI-EXPANDED, SSCI, A&HCI, ESCI Timespan=All years

#7	#3 AND #2 AND #1<br>Refined by: LANGUAGES: (ENGLISH) AND DOCUMENT TYPES: (ARTICLE) AND [excluding] WEB OF SCIENCE CATEGORIES: (FISHERIES OR PLANT SCIENCES OR ORTHOPEDICS OR TRANSPORTATION OR BIOPHYSICS OR NANOSCIENCE NANOTECHNOLOGY OR CHEMISTRY ANALYTICAL OR GEOSCIENCES MULTIDISCIPLINARY OR OCEANOGRAPHY OR PARASITOLOGY OR ELECTROCHEMISTRY OR CHEMISTRY PHYSICAL OR ENGINEERING
	MECHANICAL OR ANESTHESIOLOGY OR DERMATOLOGY OR FOOD SCIENCE TECHNOLOGY OR VETERINARY SCIENCES OR ENGINEERING CIVIL OR ENERGY FUELS OR AGRICULTURE DAIRY ANIMAL SCIENCE OR ENGINEERING ENVIRONMENTAL OR COMPUTER SCIENCE INTERDISCIPLINARY APPLICATIONS OR WATER RESOURCES OR MATHEMATICAL COMPUTATIONAL BIOLOGY OR ECONOMICS OR ZOOLOGY OR CONSTRUCTION BUILDING TECHNOLOGY OR ENGINEERING CHEMICAL OR PHYSICS APPLIED OR PHYSICS CONDENSED MATTER OR STATISTICS PROBABILITY OR BIODIVERSITY CONSERVATION OR TRANSPORTATION SCIENCE TECHNOLOGY OR EVOLUTIONARY BIOLOGY OR MARINE FRESHWATER BIOLOGY OR FORESTRY OR ENGINEERING ELECTRICAL ELECTRONIC OR GREEN SUSTAINABLE SCIENCE TECHNOLOGY OR MATERIALS SCIENCE MULTIDISCIPLINARY OR MATHEMATICS INTERDISCIPLINARY APPLICATIONS)<br>Indexes=SCI-EXPANDED, SSCI, A&HCI, ESCI Timespan=All years

Table S1.c: Search string from Embase:

No.	Query
#12	(('air pollutant'/exp OR 'air pollution'/exp OR 'black carbon'/exp OR 'carbon monoxide'/exp OR 'lead'/exp 'nitrogen oxide'/exp OR 'ozone'/exp OR 'particulate matter'/exp OR 'pm10 exposure'/exp OR 'pm2.5 exp 'sulfur oxide'/exp) OR ('ambient air pollut*':ab,ti OR 'outdoor air pollut*':ab,ti OR pm10:ab,ti OR pm2·5:a 'recreational game'/exp OR 'jogging'/exp OR 'leisure'/exp OR 'running'/exp OR 'sport'/exp OR 'walking'/e 'outdoor physical activi*':ab,ti OR 'physical activit*':ab,ti)) AND (('cardiovascular disease'/exp OR 'cardiov disease'/exp OR 'morbidity'/exp OR 'mortality'/exp OR 'vascular disease'/exp) OR ('adverse effect*':ab,ti outcome*':ab,ti OR 'cardiovascular disorder*':ab,ti OR 'cardiovascular event*':ab,ti OR 'cardiovascular ou system':ab,ti OR cvd:ab,ti OR 'vascular event*':ab,ti OR 'vascular outcome*':ab,ti)) AND [english]/lim AND
#11	(('air pollutant'/exp OR 'air pollution'/exp OR 'black carbon'/exp OR 'carbon monoxide'/exp OR 'lead'/exp 'nitrogen oxide'/exp OR 'ozone'/exp OR 'particulate matter'/exp OR 'pm10 exposure'/exp OR 'pm2.5 exp 'sulfur oxide'/exp) OR ('ambient air pollut*':ab,ti OR 'outdoor air pollut*':ab,ti OR pm10:ab,ti OR pm2·5:a 'recreational game'/exp OR 'jogging'/exp OR 'leisure'/exp OR 'running'/exp OR 'sport'/exp OR 'walking'/e 'outdoor physical activi*':ab,ti OR 'physical activit*':ab,ti)) AND (('cardiovascular disease'/exp OR 'cardiov disease'/exp OR 'morbidity'/exp OR 'mortality'/exp OR 'vascular disease'/exp) OR ('adverse effect*':ab,ti outcome*':ab,ti OR 'cardiovascular disorder*':ab,ti OR 'cardiovascular event*':ab,ti OR 'cardiovascular ou system':ab,ti OR cvd:ab,ti OR 'vascular event*':ab,ti OR 'vascular outcome*':ab,ti)) AND [english]/lim
#10	(('air pollutant'/exp OR 'air pollution'/exp OR 'black carbon'/exp OR 'carbon monoxide'/exp OR 'lead'/exp 'nitrogen oxide'/exp OR 'ozone'/exp OR 'particulate matter'/exp OR 'pm10 exposure'/exp OR 'pm2.5 exp 'sulfur oxide'/exp) OR ('ambient air pollut*':ab,ti OR 'outdoor air pollut*':ab,ti OR pm10:ab,ti OR pm2·5:a 'recreational game'/exp OR 'jogging'/exp OR 'leisure'/exp OR 'running'/exp OR 'sport'/exp OR 'walking'/e 'outdoor physical activi*':ab,ti OR 'physical activit*':ab,ti)) AND (('cardiovascular disease'/exp OR 'cardiov disease'/exp OR 'morbidity'/exp OR 'mortality'/exp OR 'vascular disease'/exp) OR ('adverse effect*':ab,ti outcome*':ab,ti OR 'cardiovascular disorder*':ab,ti OR 'cardiovascular event*':ab,ti OR 'cardiovascular ou system':ab,ti OR cvd:ab,ti OR 'vascular event*':ab,ti OR 'vascular outcome*':ab,ti))

#9	('cardiovascular disease'/exp OR 'cardiovascular system'/exp OR 'heart disease'/exp OR 'morbidity'/exp O disease'/exp) OR ('adverse effect*':ab,ti OR 'cardiac event*':ab,ti OR 'cardiac outcome*':ab,ti OR 'cardiov 'cardiovascular event*':ab,ti OR 'cardiovascular outcome*':ab,ti OR 'circulatory system':ab,ti OR cvd:ab,ti<br>'vascular outcome*':ab,ti)
#8	('exercise'/exp OR 'recreational game'/exp OR 'jogging'/exp OR 'leisure'/exp OR 'running'/exp OR 'sport'/<br>'cycling'/exp) OR (cycl*:ab,ti OR 'outdoor physical activi*':ab,ti OR 'physical activit*':ab,ti)
#7	('air pollutant'/exp OR 'air pollution'/exp OR 'black carbon'/exp OR 'carbon monoxide'/exp OR 'lead'/exp 'nitrogen oxide'/exp OR 'ozone'/exp OR 'particulate matter'/exp OR 'pm10 exposure'/exp OR 'pm2.5 exp<br>'sulfur oxide'/exp) OR ('ambient air pollut*':ab,ti OR 'outdoor air pollut*':ab,ti OR pm10:ab,ti OR pm2·5:a
#6	'adverse effect*':ab,ti OR 'cardiac event*':ab,ti OR 'cardiac outcome*':ab,ti OR 'cardiovascular disorder*'<br>OR 'cardiovascular outcome*':ab,ti OR 'circulatory system':ab,ti OR cvd:ab,ti OR 'vascular event*':ab,ti O
#5	'cardiovascular disease'/exp OR 'cardiovascular system'/exp OR 'heart disease'/exp OR 'morbidity'/exp O<br>disease'/exp
#4	cycl*:ab,ti OR 'outdoor physical activi*':ab,ti OR 'physical activit*':ab,ti
#3	'exercise'/exp OR 'recreational game'/exp OR 'jogging'/exp OR 'leisure'/exp OR 'running'/exp OR 'sport'/e
#2	'ambient air pollut*':ab,ti OR 'outdoor air pollut*':ab,ti OR pm10:ab,ti OR pm2·5:ab,ti
#1	'air pollutant'/exp OR 'air pollution'/exp OR 'black carbon'/exp OR 'carbon monoxide'/exp OR 'lead'/exp O 'nitrogen oxide'/exp OR 'ozone'/exp OR 'particulate matter'/exp OR 'pm10 exposure'/exp OR 'pm2.5 exp<br>'sulfur oxide'/exp

Quality Appraisal:

Table S2.a. Quality Appraisal Checklist for Cohort Studies

	**Andersen (2015) [1]**	**Liu (2015) [2]**	**Kubesch (2018) [3]**	**Elliot (2020) [4]**	**Lin (2021) [5]**
Were the two groups similar and recruited from the same population?	Yes	Yes	Yes	Yes	Yes
Were the exposures measured similarly to assign people to both exposed and unexposed groups?	Yes	Yes	Yes	Yes	Yes
Was the exposure measured in a valid and reliable way?	Yes	Yes	Yes	Yes	Yes
Were confounding factors identified?	Yes	Yes	Yes	Yes	Yes
Were strategies to deal with confounding factors stated?	Yes	No	Yes	Yes	Yes
Were the groups/participants free of the outcome at the start of the study (or at the moment of exposure)?	Yes	Yes	Yes	Yes	Yes
Were the outcomes measured in a valid and reliable way?	Yes	Yes	Yes	Yes	Yes
Was the follow up time reported and sufficient to be long enough for outcomes to occur?	Yes	Yes	Yes	Yes	Yes
Was follow up complete, and if not, were the reasons to loss to follow up described and explored?	Yes	Yes	Yes	Yes	Yes
Were strategies to address incomplete follow up utilized?	Yes	NA	Yes	Unclear	Yes
Was appropriate statistical analysis used?	Yes	Yes	Yes	Yes	Yes
Overall appraisal	Include	Include	Include	Include	Include
Moola S, Munn Z, Tufanaru C, Aromataris E, Sears K, Sfetcu R, Currie M, Qureshi R, Mattis P, Lisy K, Mu P-F. Chapter 7: Systematic reviews of etiology and risk . In: Aromataris E, Munn Z (Editors). Joanna Briggs Institute Reviewer's Manual. The Joanna Briggs Institute, 2017. Available from https://reviewersmanual.joannabriggs.org/					

Table S2.b. Quality Appraisal Checklist for Analytical Cross Sectional Studies

	**Hankey (2012) [6]**	**Marmett (2022) [7]**

Were the criteria for inclusion in the sample clearly defined?	Yes	Yes
Were the study subjects and the setting described in detail?	Yes	Yes
Was the exposure measured in a valid and reliable way?	Yes	Yes
Were objective, standard criteria used for measurement of the condition?	Yes	Yes
Were confounding factors identified?	Yes	No
Were strategies to deal with confounding factors stated?	Yes	Unclear
Were the outcomes measured in a valid and reliable way?	Yes	Yes
Was appropriate statistical analysis used?	Yes	Yes
Overall appraisal:	Include	Include
Moola S, Munn Z, Tufanaru C, Aromataris E, Sears K, Sfetcu R, Currie M, Qureshi R, Mattis P, Lisy K, Mu P-F. Chapter 7: Systematic reviews of etiology and risk . In: Aromataris E, Munn Z (Editors). Joanna Briggs Institute Reviewer's Manual. The Joanna Briggs Institute, 2017. Available from https://reviewersmanual.joannabriggs.org/		

Table S3: Summary of Findings

Author & Year	Physical Activity (Type, Duration & Measurements)	Air Pollution (Type & Measurements)	Outcome Measurements	Follow up	Findings	Inference
Andersen 2015 [27]	Most of the study subjects participated in physical activity: 54.3% participated in sports, 68.0% cycled, 73.5% gardened, and 93.0% walked. Participation in all physical activities was lower among those who died during follow-up than in the entire cohort, with the lowest participation among those who died from respiratory disease and diabetes.	Mean concentration of NO2 at the residence was 16.9 ± 5.2 µg/m3 for the cohort and 17.9 ± 5.7 µg/m3 for the subjects who died during follow-up.	Cardiovascular mortality was defined using ICD-10 codes (I00–I99).<br><br>Hypertension, MI, and stroke.	Study participants were followed for a mean of 13 years, resulting in 6,77,760 person-years.	Cox regression analysis showed that interaction of different PA with high NO2 resulted in reduction in cardiovascular mortality.<br><br>Statistically significant inverse associations were observed between participation in sports and all mortality with fully adjusted HZRs of 0.78 (95% CI: 0.73, 0.82) for total mortality, 0.78 (95% CI: 0.69, 0.88), for cardiovascular mortality.<br><br>Statistically significant inverse associations were observed between participation in cycling and all mortality with fully adjusted HZRs of 0.83 (95% CI: 0.78, 0.88) for total	Exposure to high TRAP levels did not modify the association, indicating beneficial effects of physical activity on CVD mortality.

					mortality, 0.78 (95% CI: 0.69, 0.88), for cardiovascular mortality.<br><br>Statistically significant inverse associations were observed between participation in gardening and all mortality with fully adjusted HZRs of 0.84 (95% CI: 0.79, 0.89) for total mortality, 0.82 (95% CI: 0.72, 0.93), for cardiovascular mortality.<br><br>Statistically non-significant associations were observed between participation in walking and all mortality with fully adjusted HZRs of 0.97 (95% CI: 0.88, 1.06) for total mortality, 0.88 (95% CI: 0.73, 1.07), for cardiovascular mortality.	
Elliott, et al 2020 [30]	Study states that higher overall PA results in a consistent decreased risk of MI and stroke and overall mortality. The study mentions that the higher vigorous PA was associated with a lower risk of overall CVD, MI, stroke and overall mortality.<br>In fully adjusted models, those in the highest quartile of physical activity (≥24:4 MET-h/wk) had 0.61 times the risk of MI or stroke (95% CI: 0.57, 0.66;ptrend<0:0001), 0.64 times the risk of MI (95% CI: 0.58, 0.71;ptrend<0:0001), 0.58 times the risk of stroke (95% CI: 0.52, 0.65;ptrend<0:0001),	Over time, average PM2.5 levels decreased from 17.0 ug/m3 in 1988 to 11:3 ug/m3 in 2006, whereas reported participation in overall physical activity increased from 15.4 MET-h/wk in 1988 to 20.9 MET-h/wk in 2006.	CVD risk (MI and stroke) and overall mortality	20 years of follow-up from 1988 to 2008	Analyses of the associations between 24-month average ambient PM2.5 exposure and risk of MI, stroke, and overall mortality showed a consistent increased risk of MI or stroke and overall mortality associated with increasing exposure.<br><br>The study observed that there is a consistent decreased risk of MI and/or stroke and overall mortality associated with higher PA and while there are no statistically significant	Higher PA was strongly associated with lower CVD risks at all levels of PM2.5 exposure.

	and 0.40 times the risk of death (95% CI: 0.37,0.42;ptrend<0:0001) in comparison with those in the lowest quartile of overall physical activity (<3:7MET-h=wk). Continuous analyses based on a 9 MET-h/wk greater overall physical activity were consistent, with 0.98 times the risk of MI or stroke (95% CI:0.97, 0.98), 0.98 times the risk of MI (95% CI: 0.97, 0.98), 0.97times the risk of stroke (95% CI: 0.97, 0.98), and 0.95 times the risk of death (95% CI: 0.94, 0.95).				differences in the associations between PA and incident MI or stroke, MI, stroke, or overall mortality by 24-month average ambient exposure to PM2.5.<br><br>For overall physical activity, walking, and vigorous physical activity, there was some suggestion that PM2.5 attenuated the beneficial effects of physical activity on risk of MI among the most exposed groups (14.4–16.4 ug/m3 and ≥16.5 ug/m3). No statistical evidence of difference in associations between physical activity and MI across quintiles of PM2.5 exposure (p interaction = 0.18–0.35). Less precise relationships between quartiles of vigorous physical activity and incident MI or stroke, MI, stroke, or overall mortality, because vigorous physical activity was reported by just 33% of the study participants.	
Hankey, et al., 2012 [32]	Self-reported physical activity levels averaged 77 min/week (IQR, 0–0 min/week; i.e., the 25th and 75th values are 0 min/week. Most (83.5%) of the survey participants reported being inactive (0 min/week), 5.6% reported being insufficiently active (1–150 min/week), and 10.9% reported being active (> 150 min/week;	The result of the survey showed that NOx and PM2.5 concentrations were highest near the city centre and major roadways, whereas O3 concentrations were higher in the outer-lying areas. Annual-average	Ischemic Heart Disease (IHD) mortality.	Self-reported activities and travel in almost one year (during the fall 2001 and spring 2002).	Physical inactivity was more strongly associated with IHD mortality (51 additional deaths/100,000/year overall) than were the other exposures, but IHD mortality attributable to physical inactivity was only slightly different between high- and low-	Health benefits from increased PA in the high walkability neighbourhoods may be reduced by the adverse effects of air pollution. Policy implications: Physical activity and exposure to air

	physical activity recommendations; U.S. Department of Health and Human Services 1996). Activity levels were notably lower than national averages (U.S. averages: inactive, 29%; insufficiently active, 45%; active, 26%). Study indicated that the average per capita physical activity was 50% higher in high- than in low-walkability neighborhoods (102 vs. 68 min/week). The number of nonsedentary individuals (people with > 0 min/week physical activity) was two times higher in high versus low-walkability neighborhoods (24.9% and 12.5%, respectively; $p < 0.001$).	air pollution exposure for the study population averaged 49 μg/m3 for NO2 [interquartile range (IQR), 41–60 μg/m3), 99 μg/m3 for O3 (86–112 μg/m3; annual average of 1-hr daily maximums), and 22 μg/m3 for PM2.5 (20–24 μg/m3). Mean NO2 exposures were below current ambient-air standards [U.S. EPA and California Environmental Protection Agency (CalEPA) standards, respectively: 100 and 57 μg/m3]. PM2.5 exposures were approximately 1.5 and 2 times higher than U.S. EPA (15 μg/m3) and CalEPA (12 μg/m3) long-term standards (annual arithmetic mean), respectively (California Air Resources Board 2010).			walkability neighborhoods (7 fewer IHD deaths/100,000/year in high- vs. low-walkability). The overall estimated attributable IHD mortality due to exposure to PM2.5 was smaller (30 deaths/100,000/year), but the difference between neighborhoods was slightly larger than for physical inactivity (9 more IHD deaths/100,000/year in high- vs. low-walkability).	pollution are critical aspects of planning for cleaner, health-promoting cities.

Kubesch 2018 [29]	Cycling, gardening, and walking.<br>The majority (99.2%) of the study participants reported being involved in 1 of the 4 physical activities. Of all participants, 54% participated in sports, 68% cycled, 74% gardened, and 93% walked (Table 1). Cohort members who participated in physical activities spent on average 2.4 h/wk participating in sports, 3.2 h/wk cycling, 3.0 h/wk gardening, and 4.3 h/wk walking. Cohort participants who developed MI (incident or recurrent MI) during follow-up were slightly less physically active (98.6%; all 4 physical activities combined) than those who did not develop a MI (99.2%).	Mean concentration of NO2 at residence was 18.7 lg/m3 in the total cohort and 18.9 lg/m3 in participants who developed a MI (incident or recurrent) during follow-up (Table 1). In this cohort, the cycling and walking levels at baseline were comparable for participants residing in areas with low (65%; 92%), medium (70%; 93%), and high (68%; 94%) NO2 levels. In contrast, gardening was more common in participants living in areas with low NO2 (85%) than in those in areas with medium (76%) and high (57%) NO2 concentrations.	Incident and recurrent MI; and hypertension.	Nil	Of 50635 participants without MI at baseline, 2936 developed incident MI.<br><br>We found inverse statistically significant associations between participation in sports (hazard ratio; 95% confidence interval: 0.85; 0.79–0.92), cycling (0.91; 0.84–0.98), gardening (0.87; 0.80–0.95), and incident MI, while the association with walking was statistically nonsignificant (0.95; 0.83–1.08). Recurrent MI was statistically nonsignificantly inversely associated with cycling (0.80; 0.63–1.02), walking (0.82, 0.57–1.16), and gardening (0.91; 0.71–1.18), and positively with sports (1.06; 0.83–1.35). There was no effect modification of the associations between physical activity and MI by nitrogen dioxide.	Benefits of PA in reducing the risk of incident and recurrent MI and SBP outweighs the risk associated with air pollution. This effect was stronger in low-TRAP environments.

Lin (2021) [31]	Commuting modes was categorized into three types as non-active (including public transportation, driving, and no need to commute), walking and cycling. Daily time of MVPA (hrs/day) was 8.4 (6.6) for non-active participants and was 8.5 (6.4), 8.2(6.7) for particpants involved in active -walking and cycling respectively. Daily sitting time (hrs/day) was 7.0 (2.9) for non-active and 7.7 (3.0), 7.0 (2.7) for participants in active walking category and cycling, respectively.	PM2.5. Satellite-based spatiotemporal model was used to estimate monthly environmental PM2.5 concentration in China from 2007 and 2008 to 2015 for each participant The PM 2.5 concentration (micrograms/m3) was 63.1 (13.2) for non-active participants and for those actively walking it was 72.4 (14.2) and for participants involved in cycling 71.9 (15.8)	CVD incidence (incident stroke, non-fatal acute MI, or death from circulatory diseases) and CHD.	China MUCA and InterASIA cohorts were followed up twice during 2007–2008 and 2012–2015, and CIMIC cohort was followed up once during 2012–2015.	Compared to non-active commuters under higher PM2.5 level, taking active commuting modes showed no beneficial effects on either CVD incidence or all-cause mortality, with the HRs (95% CIs) of 1.02 (0.91–1.15) for walking commuters and 0.87 (0.73–1.03) for cycling commuters in CVD incidence, and 0.91 (0.81–1.02) and 0.98 (0.84–1.15) in all-cause mortality. Compared to participants exposed to higher PM2.5 concentration, those in lower PM2.5 level had lower risk of CVD incidence and all-cause mortality. Cycling commuters had 40% and 58% lower risk of CVD and all-cause death compared to non-active commuters under lower PM2.5 level. Significant multiplicative interaction of commuting mode and PM2.5 level was observed in all-cause mortality (P for interaction < 0.001). The lowest mortality risk was observed in cycling participants exposed to lower level of PM2.5.	Active commuting modes by walking and cycling were associated with lower risks of CVD and all-cause mortality, as well as postponement of incident CVD and death but under lower level of PM2.5 . long-term exposure to high level of PM2.5 could counteract the cardiovascular benefits attributable to choosing active commuting modes.

Marmett (2022) [33]	The commuting variable showed higher active com-<br>muting in the outdoor group compared to untrained<br>and indoor ($p < 0.001$). Both indoor and outdoor groups demonstrated a lower inhalation rate ($p = 0.0327$). and higher physical activity levels ($p < 0.001$) compared to untrained individuals	The outdoor group had higher exposure to O_3 concentration compared to untrained (untrained group: 14.73 ug/m3/8 h; indoor group: 16.41 ug/m3/8h; outdoor group: 26.85 ug/m3 /8 h) ($p = 0.0442$). The concentration of NO_2 did not differ among groups (untrained group: 20.64 ug/m3/24h; indoor group: 19.44 ug/m3 /24 h; outdoor group: 20.48 ug/m 3 /24 h) ($p = 0.6118$).<br>The air pollutants concentration did not differ among the seasons. There was no loss of filters during the study.<br>Among the 7 areas wherein the participants live and exercise the concentration of O_3 ($p = 0.6762$) and NO_2 ($p = 0.8738$) did not differ,	<u>Cardiorespiratory fitness:</u> This study used the VO2peak as a measure of cardiorespiratory fitness<br><br><u>The Lipid accumulation product index (LAP) index</u> includes variables WC and triglycerides blood levels. (LAP=(WC(cm)-65)*TG(mmol/L);<br><br><u>Environment health risk:</u> The study investigated the impact of O_3 and NO_2 exposure on health risk based on the risk quotient, calculated using the potential intake dose and the reference dose.	NIL	Modelling was done based on O_3 exposure (model 1 representing a lower exposure and model 2 a<br>higher exposure), the concentration of O_3 had a weak positive linear correlation with risk quotient in the highest O_3 exposure model ($r = 0.3722$, $p = 0.003$), in model 1 no association was detected ($r = -0.066$, $p = 0.615$). A weak positive linear correlation was observed in model 2 between O_3 concentration and MET ($r = 0.2955$, $p = 0.020$), while in model 1 no association was observed ($r = 0.1124$, $p = 0.396$). O_3 and NO_2 concentration had no correlation in Model 1 ($r = -0.1394$, $p = 0.292$) and in model 2 ($r = 0.1025$ $p=0.4316$). Also, LAP values had a moderate negative linear correlation with VO 2peak in model 1 $r = -0.6547$, $p < 0.001$) and in model 2<br>($r = -0.5339$, $p < 0.001$) .	The study concluded that physically active individuals might have a lower risk of developing cardiovas-cular and metabolic diseases despite the higher O_3 concentration exposure, and the exposure during exercise did not represent an additional health risk.
Wen-Te Liu 2015 [28]	Walking as a commuting mode and 1 hour for commuting in the morning between 0900 -1000h.<br><br>Pearson's correlation analysis showed that PM2.5 was significantly ($p < 0.05$) correlated with TVOCs in the walking mode ($r = 0.73$), the bus mode ($r = 0.61$) and the car mode ($r = 0.59$). PM10	PM 2.5 and PM 10 were measured continuously using a personal dust monitor.<br><br>The levels of PM10, PM2.5 and TVOCs in the walking mode were 1.6-fold, 1.9-fold and 1.8-fold higher than	Three repeated measures of HRV indices were recorded using an electrocardiograph y monitor.<br><br>One-hour electrocardiograph y monitoring using a PacerCorder 3-channel device. A complete 5-min segment of the NN	Three repeated measures of 1 hour, HRV indices and mass concentration s of PM10 and PM2.5 were recorded.	Models showed that decreased HRV indices were significantly associated with increased PM10, PM2.5 and TVOC levels. Decreased HRV indices were more strongly associated with PM2.5 than with TVOCs and PM10. The greatest effect of PM2.5 on the	Walking was significantly linked with lower HRV indices due to the positive correlation between the walking mode and exposure to air pollution

	showed a weak correlation ($p < 0.10$) with PM2.5 ($r = 0.46$) and TVOCs ($r = 0.46$) in the subway mode. There were no significant differences in temperature and humidity between the four commuting modes.	those in the subway mode, respectively. The levels of these pollutants in the walking mode were approximately 1.5-fold and 1.3-fold higher than those in the car and bus modes, respectively. In accordance with the air pollution levels, the SDNN and r-MSSD values in the walking mode were 23 and 37% lower than in the subway mode, 17 and 29% lower than in the car mode and 12 and 21% lower than in the bus mode, respectively. The climate conditions were moderately pleasant, with a temperature range of 18.2 to 25.5 ◦C and a relative humidity ranging from 58.2 to 83.0%. The mean noise levels remained under 90 dBA in all of the commuting modes.	interval was taken for the HRV indices analysis, including the standard deviation of the normal-to-normal (NN) intervals (SDNN) and the square root of the mean of the sum of the squares of differences between adjacent NN intervals (r-MSSD). A total of 36 5-min HRV indices were recorded for each student during the three repeated measurements (12 segments for each measurement) for data analysis. A 4320 5-min segments of HRV indices from the 120 students in our data analysis.		SDNN was observed among the subjects in the walking mode whereas small decreases in the SDNN with PM2.5 exposure were observed among the subjects in the subway mode. A similar pattern was found in the association between PM2.5 exposure and decreased r-MSSD values. Two-pollutant mixed-effects models were performed for PM2.5 and the TVOCs; only PM2.5 was significantly associated with a decreased SDNN (-9.7%, 95% confidence interval = −13.2, −6.2%) and r-MSSD (−8.1%, 95% confidence interval = −10.5, −5.7%) after adjusting for these variables among the subjects in the walking mode. We also performed two pollutant mixed-effects models for PM10 and PM2.5 for the subjects in the walking mode and found that only PM2.5 was significantly associated with a decreased SDNN (−10.6%, 95% confidence interval = −12.9, −8.3%) and r-MSSD (−9.3%, 95% confidence interval = −11.7, −6.9%.	

Abbreviations: *CHD: Coronary heart diseases; China MUCA: China Multi-Center Collaborative Study of Cardiovascular Epidemiology; CI: Confidence Interval; CIMIC: Community Intervention of Metabolic Syndrome in China and Chinese Family Health Study; CVD: Cardio Vascular Diseases; BMI: Basal metabolic rate; h: hours; HZR: Hazard Ratio; HR: Heart rate; HRV: Heart Rate Variability; ICD-10; International Classification of Diseases, 10th Revision; IHD: Ischemic Heart Disease; InterASIA: International Collaborative Study of Cardiovascular Disease in Asia; IPAQ: International physical activity questionnaire—Short Form; LAP: Lipid accumulation product index; MET: —metabolic equivalent of task; MI: Myocardial Infarction; NO_2: Nitrogen dioxides; NOx: Nitrogen Oxides; O3: Ozone; PA: Physical Activities; PM: Particulate Matter; RQ: risk quotient; SBP: Systolic Blood Pressure; TG: Triglycerides; TRAP: Traffic Related Air Pollution; VO_2peak: peak oxygen uptake; WC: waist circumference; LAP: Lipid Accumulation Product.*

References:

[1] Andersen ZJ, De Nazelle A, Mendez MA, et al. A study of the combined effects of physical activity and air pollution on mortality in elderly urban residents: the Danish Diet, Cancer, and Health Cohort. *Environmental health perspectives* 2015; 123: 557–563.

[2] Liu W-T, Ma C-M, Liu I-J, et al. Effects of commuting mode on air pollution exposure and cardiovascular health among young adults in Taipei, Taiwan. *International journal of hygiene and environmental health* 2015; 218: 319–323.

[3] Kubesch NJ, Therming Jørgensen J, Hoffmann B, et al. Effects of leisure-time and transport-related physical activities on the risk of incident and recurrent myocardial infarction and interaction with traffic-related air pollution: A cohort study. *Journal of the American Heart Association* 2018; 7: e009554.

[4] Elliott EG, Laden F, James P, et al. Interaction between Long-Term Exposure to Fine Particulate Matter and Physical Activity, and Risk of Cardiovascular Disease and Overall Mortality in US Women. *Environmental health perspectives* 2020; 128: 127012.

[5] Lin Y, Yang X, Liang F, et al. Benefits of active commuting on cardiovascular health modified by ambient fine particulate matter in China: A prospective cohort study. *Ecotoxicology and Environmental Safety* 2021; 224: 112641.

[6] Hankey S, Marshall JD, Brauer M. Health impacts of the built environment: within-urban variability in physical inactivity, air pollution, and ischemic heart disease mortality. *Environmental health perspectives* 2012; 120: 247–253.

[7] Marmett B, Carvalho RB, Nunes RB, et al. Exposure to O3 and NO2 in physically active adults: an evaluation of physiological parameters and health risk assessment. *Environ Geochem Health*. Epub ahead of print 5 January 2022. DOI: 10.1007/s10653-021-01194-5.
